# Supplementary material for: Probing nuclear interactions à la Rutherford: insights on 4He from α scattering
Source: Nat Commun. 2026 Jun 26;17:8003. doi: 10.1038/s41467-026-74761-y (PMC13451439; doi:10.1038/s41467-026-74761-y)
Supplement: Supplementary file 1 [file 41467_2026_74761_MOESM1_ESM.pdf]

Supplementary Information

Probing nuclear interactions à la Rutherford: Insights on ^{4}He from α scattering

F. Cappuzzello[1,2], V. Soukeras[1,2*], S. Bacca[3,4], D. Carbone[2], M. Cavallaro[2], L. C. Chamon[5], I. Lombardo[1,6], G. Orlandini[7,8], M. Viviani[9], C. Agodi[2], H -W. Becker[10], G. A. Brischetto[1,2], S. Calabrese[2], C. Ciampi[11], M. Cicerchia[12,13], M. Cinausero[14], I. Ciraldo[1,2], D. Dell'Aquila[15,16], M. Fisichella[2], C. Frosin[11], A. Hacisalihoglu[2,17], M. Hilcker[18], A. Kievsky[9], Y. Kucuk[19,20], T. Marchi[14], O. Sgouros[1,2], A. Spatafora[1,2], D. Torresi[2], M. Vigilante[15,16], A. Yildirim[20]

[1]Dipartimento di Fisica e Astronomia "Ettore Majorana", Università di Catania, Catania, Italy.
[2]INFN – Laboratori Nazionali del Sud (LNS), Catania, Italy.
[3]Institut für Kernphysik, Johannes Gutenberg-Universität Mainz, Mainz, Germany.
[4]Helmholtz-Institut Mainz, Johannes Gutenberg-Universität Mainz, Mainz, Germany.
[5]Departamento de Física Nuclear, Instituto de Física da Universidade de São Paulo, São Paulo, Brazil.
[6]INFN – Sezione di Catania, Catania, Italy.
[7]Dipartimento di Fisica, Università di Trento, Trento, Italy.
[8]INFN - Trento Institute for Fundamental Physics and Applications (TIFPA), Trento, Italy.
[9]INFN – Sezione di Pisa, Pisa, Italy.
[10]Ruhr-Universitat Bochum, Bochum, Germany.
[11]INFN – Sezione di Firenze, Firenze, Italy.
[12]Dipartimento di Fisica e Astronomia, Università di Padova, Padova, Italy.
[13]INFN – Sezione di Padova, Padova, Italy.
[14]INFN – Laboratori Nazionali di Legnaro (LNL), Legnaro, Italy.

[15]Dipartimento di Fisica "Ettore Pancini", Università degli Studi di Napoli "Federico II", Napoli, Italy.
[16]INFN – Sezione di Napoli, Napoli, Italy.
[17]Institute of Natural Science, Karadeniz Teknik Universitesi, Trabzon, Turkey.
[18]Department of Physics, Institute for Nuclear Physics, Technische Universität Darmstadt, Darmstadt, Germany.
[19]Turkish Accelerator and Radiation Laboratory (TARLA), Gölbaşı/Ankara, Turkiye.
[20]Akdeniz University, Antalya, Turkey.

*Corresponding author(s). E-mail(s): vasileios.soukeras@lns.infn.it;

Contents

1 Supplementary Methods **3**
- 1.1 Reaction Theory Details . . . 3
  - 1.1.1 Adopted potentials in the reaction theory . . . 5
- 1.2 Ab-initio theory details . . . 6
  - 1.2.1 Nuclear densities: bound-state vs continuum approach . . . 7

2 Supplementary Figures **10**

3 Supplementary Tables **16**

Supplementary References **17**

1 Supplementary Methods

1.1 Reaction Theory Details

We have performed cross section calculations for the α-α elastic and inelastic scattering in the framework of the coupled channel quantum scattering theory [1]. A set of two coupled equations is written for each component l of the angular momentum of the relative motion of one α with respect to other α as

$$\begin{aligned}\left[-\frac{\hbar^2}{2\mu}\frac{d^2}{dR^2}+\frac{l(l+1)}{2\mu R^2}+U_1(R)\right]\psi_l^{(1)}+U_{\rm coup}(R)\psi_l^{(2)} &= E_{\rm cm}\,\psi_l^{(1)}, \\ \left[-\frac{\hbar^2}{2\mu}\frac{d^2}{dR^2}+\frac{l(l+1)}{2\mu R^2}+U_2(R)\right]\psi_l^{(2)}+U_{\rm coup}(R)\,\psi_l^{(1)} &= (E_{\rm cm}-E_X)\,\psi_l^{(2)},\end{aligned} \tag{1}$$

where the wavefunctions for the elastic and inelastic channels have the following asymptotic behavior:

$$\begin{aligned}\psi_l^{(1)}(R\to\infty) &= \tfrac{i}{2}\left[H_l^- - S_l^{(1)}H_l^+\right], \\ \psi_l^{(2)}(R\to\infty) &= -\tfrac{i}{2}S_l^{(2)}H_l^+,\end{aligned} \tag{2}$$

and $H_l^{\pm}$ represent the Coulomb Hankel functions for the respective channel. Since we deal with identical particles of spin zero, we must consider only even values of l. U_1 is the diagonal nucleus-nucleus optical potentials in the initial state interaction (ISI), which is the same as the final state interaction (FSI) only in the case of the elastic scattering process. For the inelastic channel, the U_2 optical potential represents the FSI, while $U_{\rm coup}$ is the coupling potential.

We assume the following model for the diagonal part of the ISI and FSI interactions in Eq. (1)

$$U_j(R) = V_j^{(C)}(R) + N_{Rj}V_j^{(N)}(R) + iN_{Ij}V_j^{(N)}(R),\ j=1,2 \tag{3}$$

where $V^{(C)}$ and $V^{(N)}$ are the Coulomb and nuclear potentials. N_{Rj} and N_{Ij} are normalization factors for the real and imaginary parts of the optical potential for the elastic channel ($j=1$) that we use in our analyses. The corresponding values are dependent on the angular momentum l, as explained below. For the inelastic channel, we set $N_{R2}=1$ and $N_{I2}=0$.

We obtain the Coulomb and nuclear potentials for the diagonal parts of the interaction within the double folding model, namely through

$$V_j(\vec{R}) = \int \rho_{0_1^+}(\vec{r}_1)\,\rho_{0_j^+}(\vec{r}_2)\,v_{NN}(\vec{R}-\vec{r}_1+\vec{r}_2)\,d\vec{r}_1\,d\vec{r}_2, \tag{4}$$

where $\rho_{0_1^+}(\vec{r}_1)$ is the ^{4}He density in its ground-state 0_1^+. Here, $\rho_{0_j^+}(\vec{r}_2)$ is the density of either the 0_1^+ or the 0_2^+ when dealing with $U_1(R)$ and $U_2(R)$, respectively. For

the coupling potential, $U_{\rm coup}$, we adopt a similar double-folding procedure as that of Eq. (4), where the second density is this time replaced by the transition density $\rho_{0_1^+\to 0_2^+}$.

In Eq. 4, v_{NN} represents the effective nuclear plus Coulomb interactions. For such a force, we use the Brazilian Nuclear potential (BNP) [2, 3] to calculate the nuclear part of the double-folding potentials. Although the nucleon-nucleon force used in the folding is different from the Hamiltonian used in the ab-initio densities, this "inconsistency" is absorbed in the modeling intrinsic to the double–folding approach. Within this model, we adopt matter densities ($\rho = \rho_m$) in equation (4), which is related to the distribution of nucleons ρ_N as [2]

$$\rho_m(r) = \int \rho_N(|\vec{r} - \vec{r'}|)\, \rho_{m/N}(r')\, d\vec{r'}, \tag{5}$$

where $\rho_{m/N}$ is associated to the intrinsic matter density of one nucleon (which is equal the intrinsic charge density of one proton). Since the nucleon is not a point-like particle and has a finite size, the matter density of the nucleus $\rho_m(r)$ is different—through Eq. (5)—from the point-like distribution of nucleons in the nucleus $\rho_N(r)$ [2, 3].

For the case of ^{4}He, where the number of protons is equal to the number of neutrons, we assume that the matter density is twice the charge density, for both the 0_1^+ and 0_2^+ states, and adopt the experimental charge density obtained from electron scattering experiments of Ref. [4] to describe the ground state. It is important to note that the use of g.s. densities from ab-initio for the 0_1^+ state instead of that from the experimental charge does not significantly modify the diagonal double-folding potentials for either channel.

The calculation of the folding potential is performed through Fourier transform method [5–7]), which for the coupling potential reads

$$U_{\rm coup}(R) = \frac{1}{2\pi^2} \int \hat{v}_{NN}(q)\, \hat{\rho}_{0_1^+}(q)\, \hat{\rho}^{0_1^+\to 0_2^+}(q)\, J_0(qR)\, q^2\, dq, \tag{6}$$

where $J_0(x) = \sin(x)/x$ is the spherical Bessel function of order zero, $\hat{\rho}_{0_1^+}$ is the Fourier transform of the (matter or charge) 0_1^+ density,

$$\hat{\rho}_{0_1^+}(q) = 4\pi \int \rho_{0_1^+}(r)\, J_0(qr)\, r^2\, dr, \tag{7}$$

and $\hat{v}_{NN}$ is the Fourier transform of the nucleon-nucleon and Coulomb interactions. The term $\hat{\rho}^{0_1^+\to 0_2^+}$ in Eq. (6) is the Fourier transform of the matter transition density

$$\hat{\rho}_0^{g.s.\to 0_2^+}(q) = \sqrt{4\pi} \int \rho_0^{g.s.\to 0_2^+}(r)\, J_0(qr)\, r^2\, dr. \tag{8}$$

As done for the diagonal densities, we assume the matter transition density to be twice the charge transition density. This ansatz is used in Eq. (6) to obtain the nuclear and Coulomb coupling potentials, respectively. In similar form as in Eq. (5),

the transition density of charge (or matter) is related to the respective distribution of protons (or nucleons) by

$$\rho^{0_1^+\to 0_2^+}_{\text{charge or matter}}(r) = \int \rho^{0_1^+\to 0_2^+}_{\text{protons or nucleons}}(|\vec{r}-\vec{r'}|)\, \rho_{m/N}(r')\, d\vec{r'}, \tag{9}$$

Equation (9) results in the following correlation among the respective transforms

$$\hat{\rho}^{0_1^+\to 0_2^+}_{\text{charge or matter}}(q) = \hat{\rho}^{0_1^+\to 0_2^+}_{\text{protons or nucleons}}(q) \times \hat{\rho}_{m/N}(q), \tag{10}$$

where the Fourier transform for the proton finite size factor is well known from electron scattering experiments

$$\hat{\rho}_{m/N}(q) \approx \frac{1}{(1+0.0552\, q^2)^2}. \tag{11}$$

In Supplementary Figs. 3(a-c) we present the diagonal and transition matter densities obtained from the corresponding nucleon densities reported on Refs. [8–11].

1.1.1 Adopted potentials in the reaction theory

At low energies ($E_{\text{Lab}} < 34.6$ MeV), the only open reaction channel for ^{4}He + ^{4}He is the capture process with γ decay, ^{4}He(^{4}He,γ)^{8}Be, followed by the break-up of the beryllium into two α-particles. However, this process has a very small cross section. Thus, in this energy region, we can neglect the absorption of flux from the elastic channel. In this condition, the optical potential for the elastic channel must have vanishing imaginary part ($N_{I1} = 0$). In addition, all the respective elements of the S matrix must have unitary modulus. Therefore, each of these elements is characterized just by the respective phase-shift. Furthermore, considering that only a small number of waves contribute to the scattering, in this low-energy region the elastic scattering angular distribution depends on a small number of even partial waves (typically $l = 0$, 2, 4 and 6). Due to the small number of free parameters, the phase-shifts were obtained quite accurately as a function of the energy from elastic scattering data analyses.

In Supplementary Fig. 4, we show the $l = 0, 2, 4, 6$ phase-shifts and moduli of the S matrix (for the elastic channel), as a function of the energy. The experimental values in the figure were obtained in several works [12–21] and we emphasize (through pink star symbols) the data from Ref. [21] at $E_{\text{Lab}} = 53.4$ MeV and 63.9 MeV. Indeed, these energies are close to the energies of the inelastic scattering data analyzed in the present work: 53.0 MeV and 63.8 MeV. The red solid lines in Supplementary Fig. 4 represent the results obtained with the standard BNP adopting $N_{R1} = 1$ and $N_{I1} = 0$ in Eq. (3). It is clear that the standard BNP provides a quite good description of the experimental phase-shifts in the low energy region.

For energies above the threshold mentioned above, $E_{\text{Lab}} > 34.6$ MeV, the experimental phase-shifts deviate from the BNP predictions (see Supplementary Fig. 4), probably due to effects of the polarization arising from the couplings to all the open reaction channels. In addition, at these energies the modulus of S is not unitary anymore, as can be clearly observed from the experimental values presented in the

figures. These deviations can be effectively taken into account in the theoretical calculations through the renormalization of the nuclear potential, adjusting the N_{R1} and N_{I1} parameters in the $U_1(R)$ potential.

We have tuned the values of N_{R1} and N_{I1} for the elastic channel in order to reproduce, as well as possible, the phase-shifts and the modulus of S at the energies of $E_{\mathrm{Lab}} = 53.4$ MeV and $E_{\mathrm{Lab}} = 63.9$ MeV (pink star symbols in Supplementary Fig. 4). The adjustment was performed separately for each l value. The corresponding best N_{R1} and N_{I1} values are provided in Supplementary Table 2. The respective theoretical results for phase-shifts and moduli of S are indicated by the blue dashed lines in Supplementary Fig. 4, which show a reasonable agreement with the experimental values in the region $50 \leq E_{\mathrm{Lab}} \leq 65$ MeV. Note, in Supplementary Table 2, that the N_{R1} values are only slightly different from 1, except in the case of $l = 6$. Observe also that the N_{I1} values are small, about $N_{I1} = 0.1$. This indicates a small but non negligible overall polarization and absorption effect induced by the reaction channels not explicitly included in our coupled channel equations.

1.2 Ab-initio theory details

Theoretical approaches in which nuclear properties are derived from virtually exact solutions of the Schrödinger equation for nucleons interacting via two- and three-body forces—commonly known as ab initio methods—have been extensively tested on light nuclei. Besides the famous benchmark [22] for the α-particle ground-state, ab-initio theory has been recently challenged by the transition form factor $F_M(0_1^+ \to 0_2^+)$ of the α-particle. We describe the various calculations below in chronological order, as they constitute the motivation for the present work.

The first calculation, performed in 2004 by Hiyama et al. [8], achieved a good agreement with experimental electron scattering data for F_M, despite employing a relatively simple Hamiltonian. A subsequent study by Bacca et al. [23] in 2013, using realistic chiral effective field theory (χEFT) potentials, significantly overestimated the experimental values. This discrepancy raised questions about either the accuracy of realistic Hamiltonians or the reliability of the experimental data. The latter possibility was recently ruled out by a high-precision measurement by Kegel et al. [24], which confirmed the earlier experimental results with much improved accuracy. As a result, the tension with the realistic theory has intensified, prompting a wave of new theoretical calculations—some of which are consistent with the experimental data [10, 11, 25], while others continue to show disagreement [26].

Michel et al. [25], in a calculation that neglected three-body forces managed to reproduce the Kegel data by tuning the Hamiltonian to fit the 0_2^+ resonance position to experiment, $E_r \approx 0.4$ MeV [27]. However, a mistake was subsequently found [28], leading to the real energy of the Gamow pole to be 0.12 MeV. Meißner et al. [11] obtained the correct description of the Kegel et al. data in the framework of nuclear lattice EFT adopting a simple Hamiltonian, which is based on the SU(4) isospin symmetry. The parameter of this Hamiltonian were calibrated on a variety of data including medium-mass nuclei, excluding the position of the 0_2^+ resonance, which nevertheless is

correctly reproduced. However, the Fourier transform of the obtained transition density in coordinate space (see Fig. 2 of Ref. [11]) is about 20% higher than the form factor presented in Fig. 1 of the same reference.

These facts expose the unexpected situation in which possibly realistic Hamiltonians did not reproduce the monopole transition strength, while simpler Hamiltonians did. However, another calculation by Viviani et al. [10] reproduced F_M with realistic chiral EFT Hamiltonians—though different from those used in Ref. [23]— but found a low E_r. Moreover, Yin et al. [26] performed a calculation with no-core shell model using the Daejeon16 potential, a different realistic Hamiltonian, obtaining a result very similar to [23], therefore not consistent with experimental data. Finally, the 0_2^+ state has been subject of a recent benchmark study using sophisticated techniques to solve the four-body problem, namely the solution of the Faddeev-Yakuboski, the Alt-Grassberger-Sandhas equations and the hyperspherical harmonics method [29]. Without considering the Coulomb interaction this state results bound and very close to the p-^{3}H threshold. In this case the 0_1^+ and 0_2^+ can be identified as the two Efimov four–body states attached to the lowest three-body Efimov state, in this case the triton [30]. Including the Coulomb interaction the 0_2^+ state crosses the p-^{3}H threshold and moves into the continuum becoming a resonance [29].

Given the puzzling situation, we have to find other ways to probe the 0_2^+ state in the α-particle. Ab-initio calculations can deliver a density for 0_1^+ and 0_2^+, as well as a transition density from 0_1^+ to 0_2^+. They can be used as input in reaction theory to construct potentials within the double folding model, and then be compared with experimental data. In this work, we collect densities from the following calculations: Hiyama et al. [8], Meißner et al. [11], Bacca et al. [9, 23], and Viviani et al. [10]. The first two calculations use simple Hamiltonians, while the second two use realistic chiral EFT interactions with the same two-body force [31, 32] at next-to-next-to-next-to-leading order (N3LO) with cutoff $\Lambda = 500$, and a three-body force derived at next-to-next-to leading order (N2LO) [33]. The latter contains two free parameters, namely the coupling constant multiplying the one-pione-exchange (c_D) and that multiplying the contact term (c_E). These two parameters are different in Ref. [9] and [10]: c_D and c_E are fixed to reproduce ^{3}H and ^{4}He binding energies following [34] in [9], while they are fit to reproduce the ^{3}H binding energy and the Gamow-Teller matrix element of tritium beta decay [35] in [10]. Both calculations [23] and [10] are performed using the hyperspherical harmonics (HH) expansion method [10, 36–40], where one can construct either bound states or continuum states, as explained below.

1.2.1 Nuclear densities: bound-state vs continuum approach

From the point of view of ab-initio calculations, the nuclear densities used in Sect. 1.1 are defined as

$$\rho_{a,b}(r) = \langle \Psi_a | \sum_{j=1}^{4} \delta(\boldsymbol{r}_j - \boldsymbol{r}) | \Psi_b \rangle \ , \tag{12}$$

where $a, b = 0_1^+, 0_2^+$ and $\Psi_{0_1^+}$ ($\Psi_{0_2^+}$) is the ^{4}He ground state (first excited state) wave function.

For the ground state, the wave function can be obtained from an expansion over a complete set of orthonormal square-integrable basis states Φ_ν as

$$\Psi_{0_1^+} = \sum_{\nu=1}^{N} a_\nu \Phi_\nu \ . \tag{13}$$

The Hamiltonian represented on this basis $M_{\nu,\nu'} = \langle \Phi_\nu | H | \Phi_{\nu'} \rangle$ is then used in the eigenvalue problem $\sum_{\nu'} M_{\nu,\nu'} a_{\nu'} = E_\nu a_\nu$, which is solved for increasing N. The lowest eigenvalue is the ^{4}He ground state energy $E_{0_1^+}$. When hyperspherical harmonics are used as the basis, this variational approach is called the HH method [10, 39, 40]. To speed up the convergence in N, but renouncing to the variational property, an effective interaction can be introduced leading to a method called EIHH [37, 38]. For infinite N, both the HH and EIHH approaches converge to the same exact result, provided that the same Hamiltonian is used.

Regarding $\Psi_{0_2^+}$, we have two choices. First, in a bound-state approximation, we can select the second lowest eigenvalue of the above eigenvalue problem to be $E_{0_2^+}$ and construct $\Psi_{0_2^+}$ with the associated coefficients a_ν.

The corresponding densities in Eq. (12) are normalizable, since $\Psi_{0_2^+}$ is written in terms of square integrable states.

However, this state is not a true bound state but a resonance, rapidly decaying in a proton and a ^{3}H nucleus. Therefore the second option is to calculate $\Psi_{0_2^+}$ in the continuum. Using the HH formulation of Refs. [10, 39, 40] we have computed the $p + {}^3$H scattering wave functions Ψ_E, where E is now the (positive) kinetic energy of the two clusters p and ^{3}H when they are well apart. The total energy of this scattering state (with respect to the ground state, and therefore corresponding to the energy transferred to the α-target) is $E_T = E + E_{th}$, where E_{th} is the threshold energy given by the difference between the ^{4}He and ^{3}H binding energies. We can also introduce the relative inter-cluster momentum q_{ic}, defined to be $q_{ic}^2/2\mu = E$, where μ is the $p + {}^3$H reduced mass. The wave function Ψ_E can be calculated using the HH method (see Ref. [10]) as well, but this time in the expansion we have to include also non-square-integrable functions, describing the motion of the $p + {}^3$H when they are far from each other. Explicitly,

$$\Psi_E = \sum_{\nu=1}^{N} a_\nu \Phi_\nu + \Psi_A \ , \tag{14}$$

where Ψ_A is the asymptotic non-square-integrable part. The first term of Eq. (14) is non zero only where the four nucleons are close to each other. When the $p + {}^3$H distance is above 20 fm, Ψ_E reduces to Ψ_A.

In the range of energies probed in our experiment, the S-wave $p + {}^3$H phase shift $\delta(E)$ has a typical resonance behavior, namely a sharp increase reaching the value of 90°. In such a case, Ψ_E tends to have the form of a "quasi-bound" state, namely to be very large in the region where the four nucleons are close to each other, much larger than Ψ_A. In a time-dependent formalism, this means that the four particles tend to stay for a long time together before the system falls apart as a $p + {}^3$H scattering state.

The short-range behavior of Ψ_E is very similar to $\Psi_{0_2^+}$, calculated as a bound state. To show this, we have calculated the following densities

$$\rho_{0_1^+,E}(r) = \langle \Psi_{0_1^+} | \sum_{j=1}^{4} \delta(\boldsymbol{r}_j - \boldsymbol{r}) | \Psi_E \rangle \ , \qquad \rho_E(r) = \langle \Psi_E | \sum_{j=1}^{4} \delta(\boldsymbol{r}_j - \boldsymbol{r}) | \Psi_E \rangle \ . \qquad (15)$$

The density $\rho_E(r)$ (see Supplementary Fig. 7 for its distribution as a function of r for different E) cannot be normalized, since $\int d^3r\ \rho_E(r) = \infty$. In fact, for large r it goes like $\rho_E(r) \to \sin^2(q_{ic}r + \delta)/r^2$. In particular, as $E \to 0.20$ MeV, the particle density for $r < 1$ fm rapidly increases, showing the formation of a quasi-bound state. In Supplementary Fig. 3(d) we compare the calculated $\rho_{0_2^+,0_2^+}(r)$ with $\alpha \times \rho_E(r)$, rescaled so that $\alpha \times \rho_E(r=0) = \rho_{0_2^+,0_2^+}(0)$. Since in our experiment, we mainly probe $p + {}^3$H energies in the region $0.35 < E < 0.65$ MeV, we have reported in the figure $\rho_{E=0.35\ \mathrm{MeV}}$, $\rho_{E=0.50\ \mathrm{MeV}}$, and $\rho_{E=0.65\ \mathrm{MeV}}$. As it can be seen by inspecting the figure, the various densities are quite similar. The $\rho_E(r)$ curves are large when $r < 1$ fm and have the same plateau as $\rho_{0_2^+,0_2^+}$, and then they decrease in a similar way. Only for large r, there would be large differences (not visible in the figure), as $\rho_{0_2^+,0_2^+}$ goes rapidly to zero, while ρ_E has the oscillating behavior mentioned above. We can observe the same similarities in $\rho_{0_1^+,0_2^+}$ and $\rho_{0_1^+,E}$. As a consequence, we can safely apply the bound-state densities to study the ^{4}He+^{4}He reaction.

2 Supplementary Figures

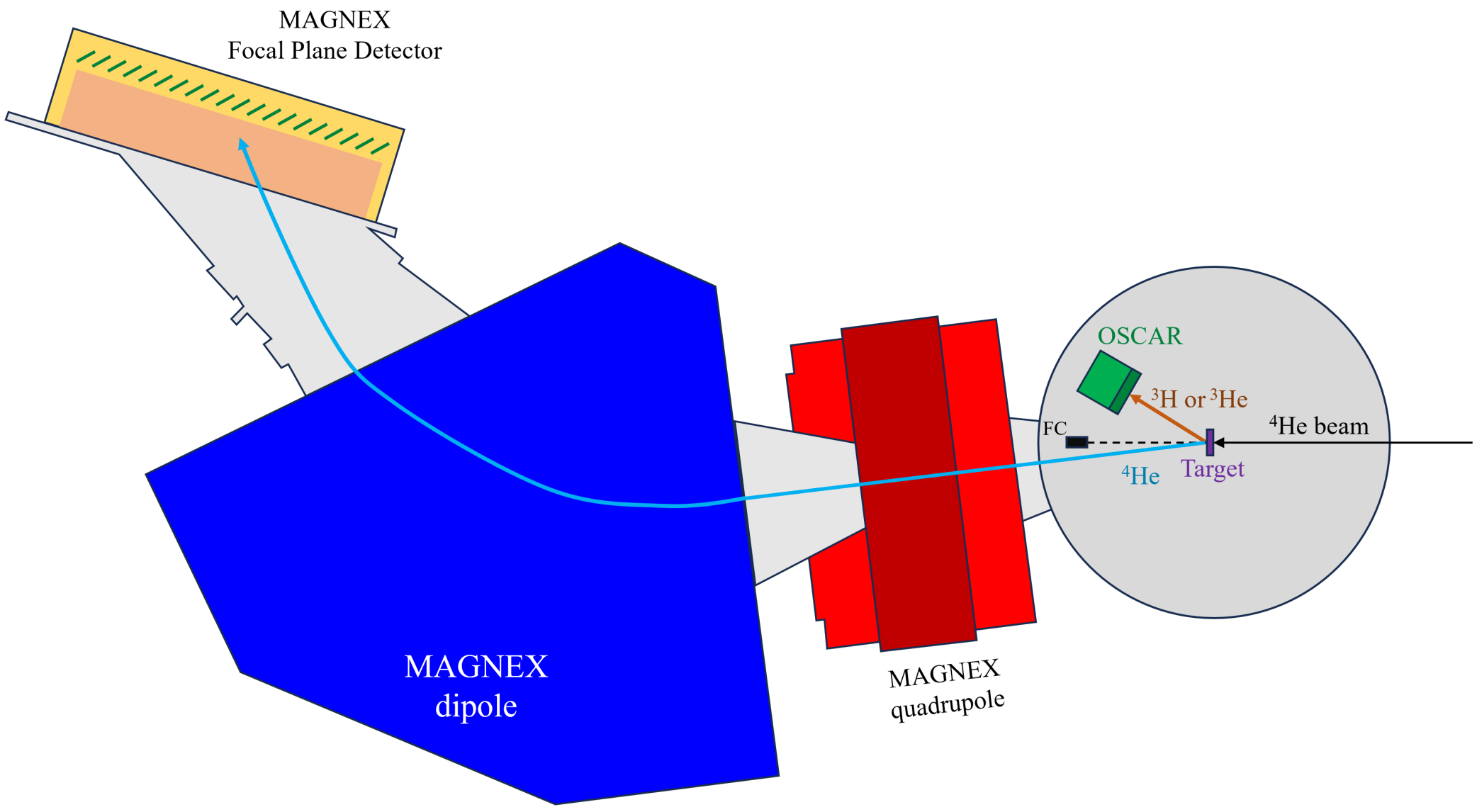

Supplementary Fig. 1 Experimental set-up. Illustration of the experimental set-up for the exclusive measurement (the scattering chamber is not in scale for a better readability). The cyan blue arrow represents a symbolic sketch of the trajectory of a ^{4}He ejectile across the MAGNEX spectrometer while the orange arrow the trajectory of ^{3}H or ^{3}He decay products detected in coincidence by the OSCAR telescope.

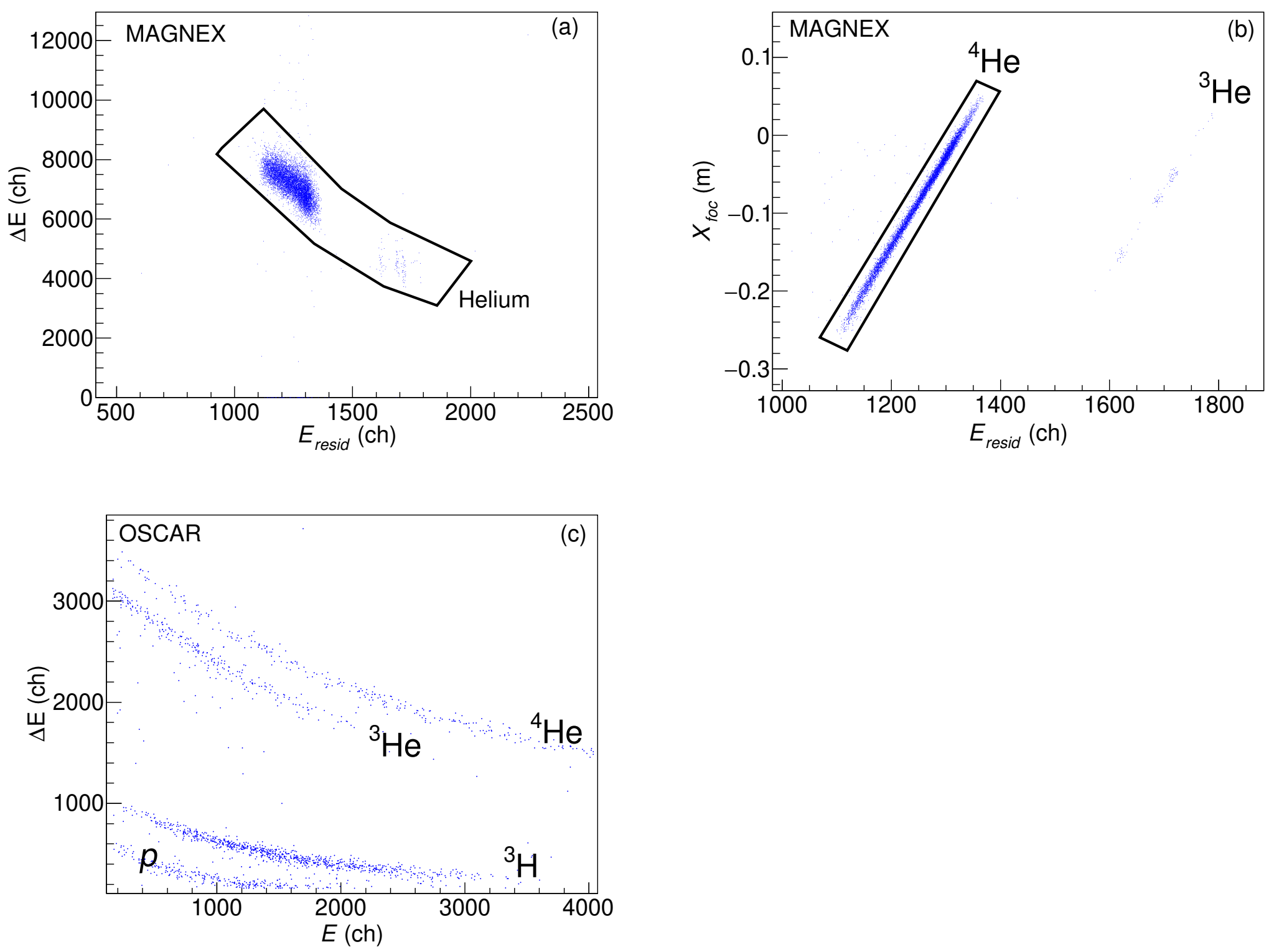

Supplementary Fig. 2 Particle identification spectra. (a) A typical ΔE versus residual energy (E_{resid}) spectrum for a single silicon detector of MAGNEX Focal Plane Detector (FPD). (b) Horizontal position (X_{foc}) versus residual energy (E_{resid}) spectrum for a single silicon detector of the MAGNEX FPD after applying the graphical selection of Z=2 (Helium) of the panel (a). (c) A typical ΔE versus residual energy (E) spectrum for a combination of a single ΔE strip correlated with a single E pad of OSCAR.

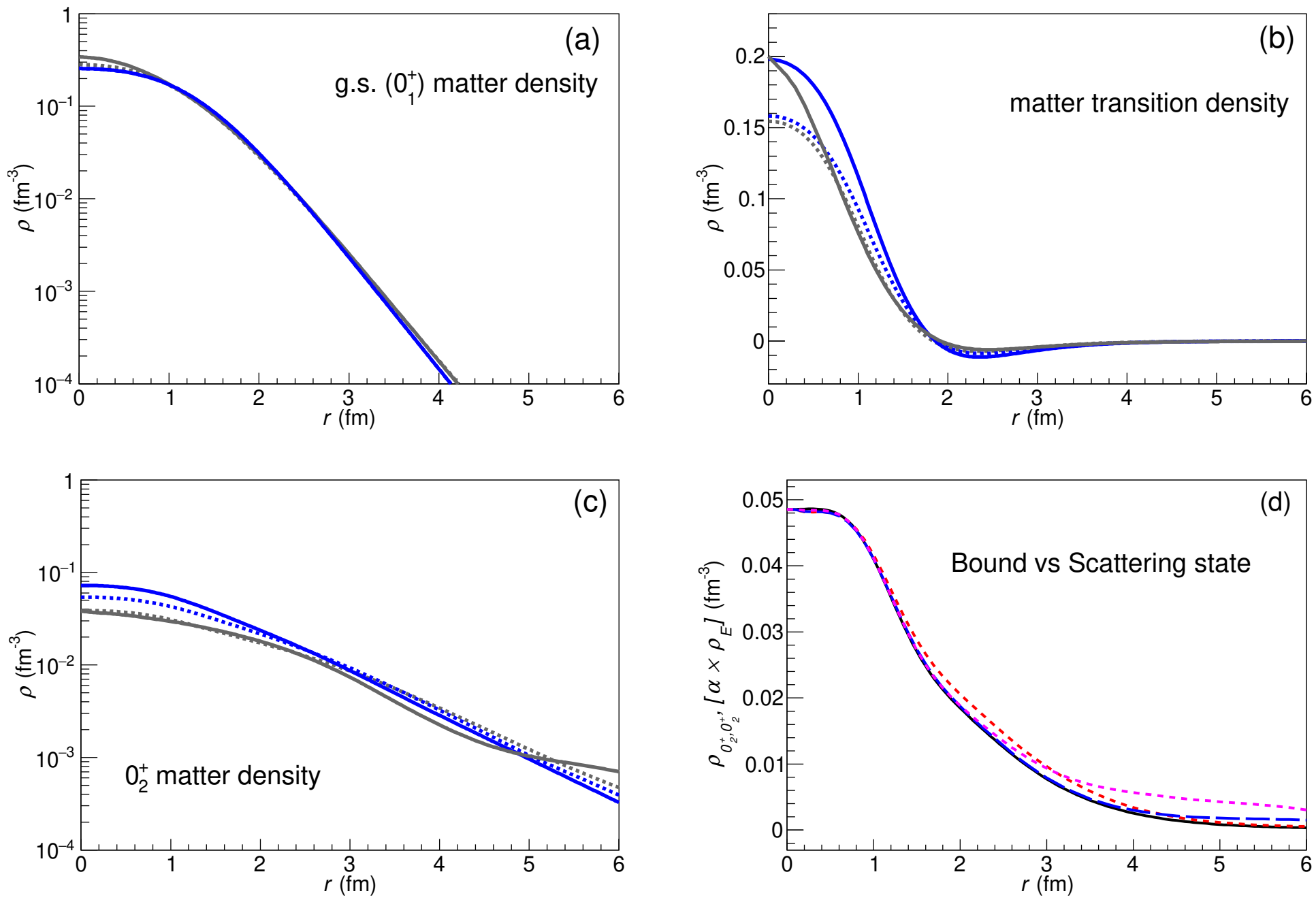

Supplementary Fig. 3 Calculated densities. (a) Matter density (ρ) for the ^{4}He g.s. derived (see section 1.1) from Refs. [9] (Bacca et al. - χEFT), [10] (Viviani et al.- χEFT), [11] (Meißner et al.) and [8] (Hiyama et al.) are depicted with the blue solid, blue dashed, grey solid and grey dashed lines, respectively. (b) Same as in (a) but, for the transition matter densities. (c) Same as in (a) but, for the 0_2^+ state. (d) Calculated density $\rho_{0_2^+,0_2^+}(r)$ denoted with the black solid line is compared with the $[\alpha \times \rho_E(r)]$, rescaled so that $\alpha \times \rho_E(r=0) = \rho_{0_2^+,0_2^+}(0)$. Calculations for $E = 0.35$ MeV, $E = 0.50$ MeV and $E = 0.65$ MeV are denoted with the red, blue and magenta dashed lines, respectively. See Methods - Calculation methodology and Supplementary Methods (1.2.1) for details.

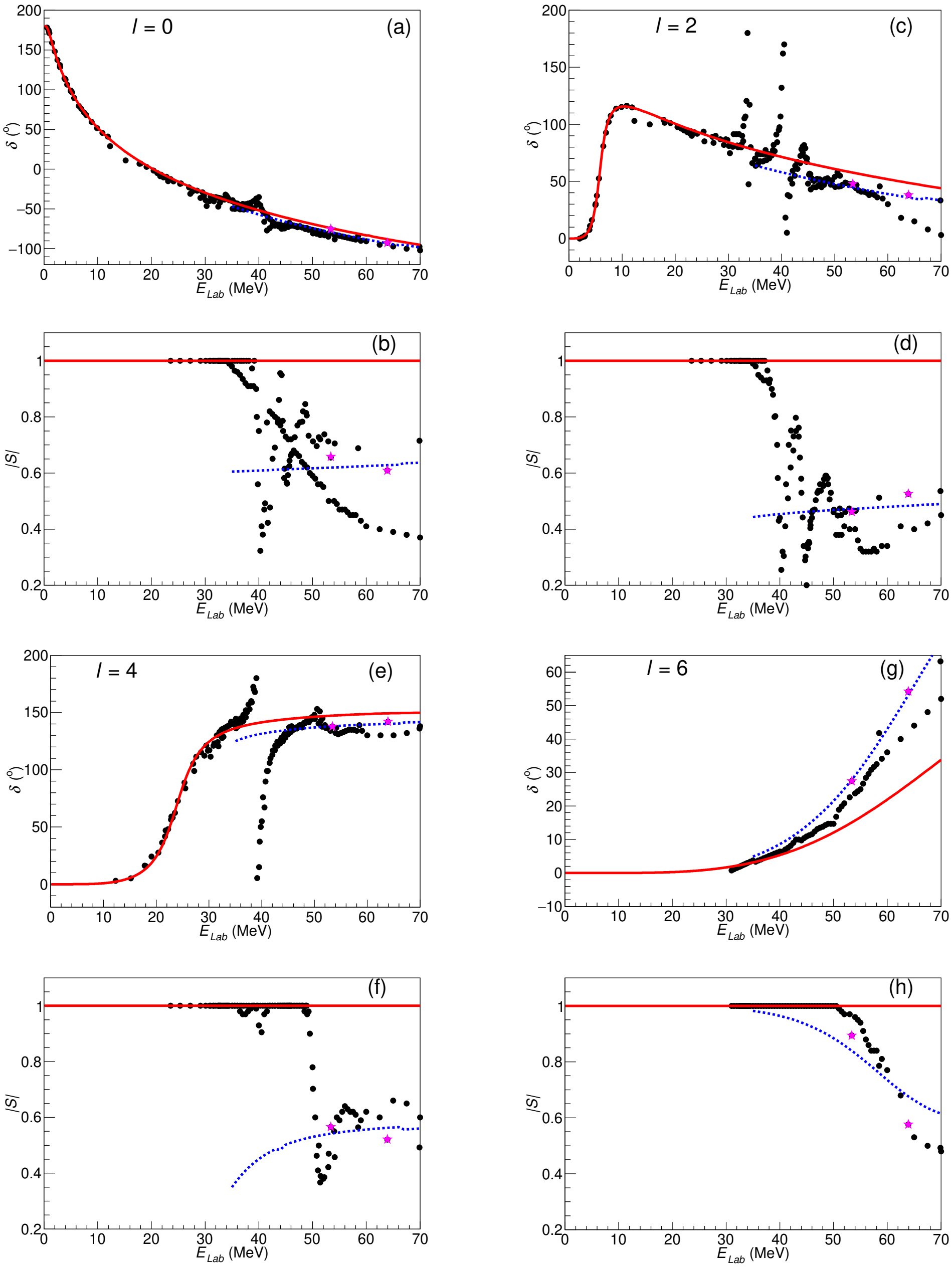

Supplementary Fig. 4 Phase-shift (δ) and modulus of the S matrix. Experimental values [12–21] for the phase-shift δ (a, c, e, g) and modulus of the S matrix (b, d, f, h), obtained for the elastic channel, as a function of the energy for $l = 0$, $l = 2$, $l = 4$ and $l = 6$, respectively. The data of Ref. [21] at 53.4 and 63.9 MeV, similar incident energies with the present analysis, are emphasized with the magenta stars. The red solid lines in the figure represent the predictions of the standard Brazilian Nuclear Potential (BNP). The blue dashed lines were obtained considering the renormalization of the BNP through the N_R and N_I values discussed in Supplementary Methods (1.1.1).

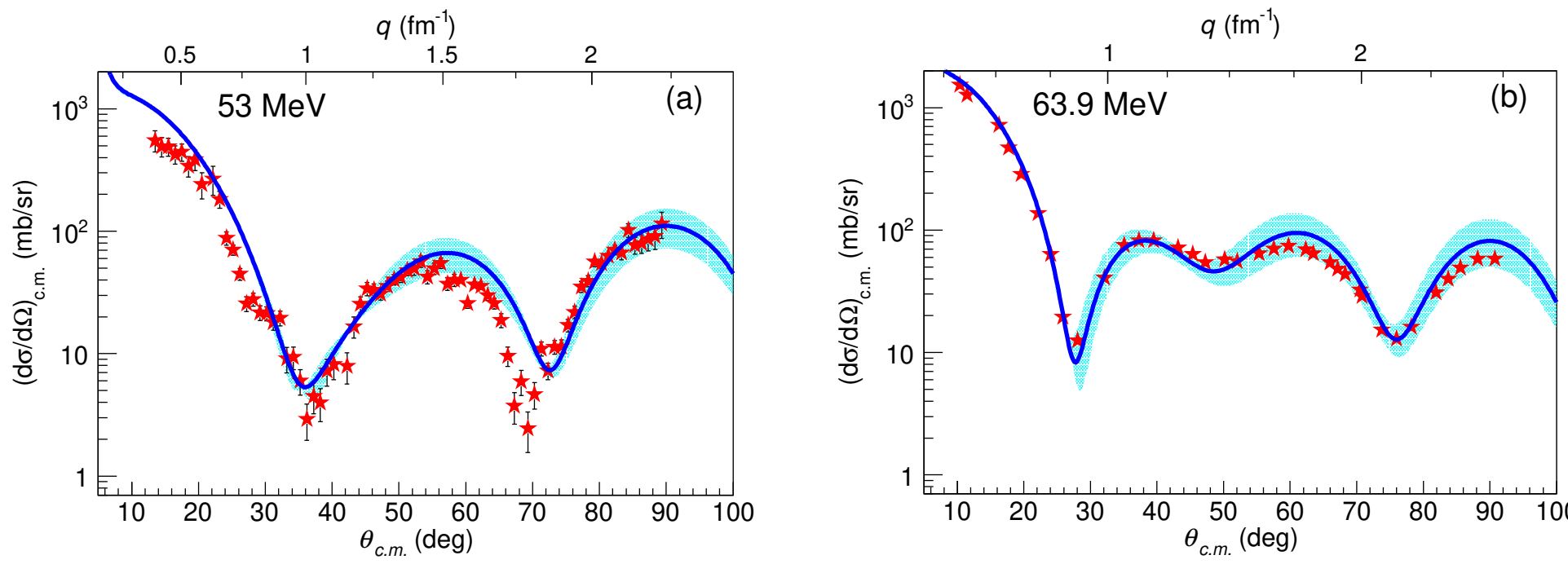

Supplementary Fig. 5 Elastic scattering angular distributions and uncertainties in the reaction modeling. (a) Experimental elastic scattering angular distribution at 53 MeV (red stars) compared with theoretical calculations taking into account a phase shift analysis using the data of Ref. [21] at 53.4 and 63.9 MeV (blue solid line). The error bars correspond to the combination of uncertainties coming from the solid angle and detection efficiency estimation, the uncertainties in the beam current and the target thickness determination, the statistical error and the background subtraction. (b) Same but for the incident energy of 63.9 MeV with experimental data from Ref. [21]. In both panels, the cyan blue band accounts for the uncertainties in the reaction modeling.

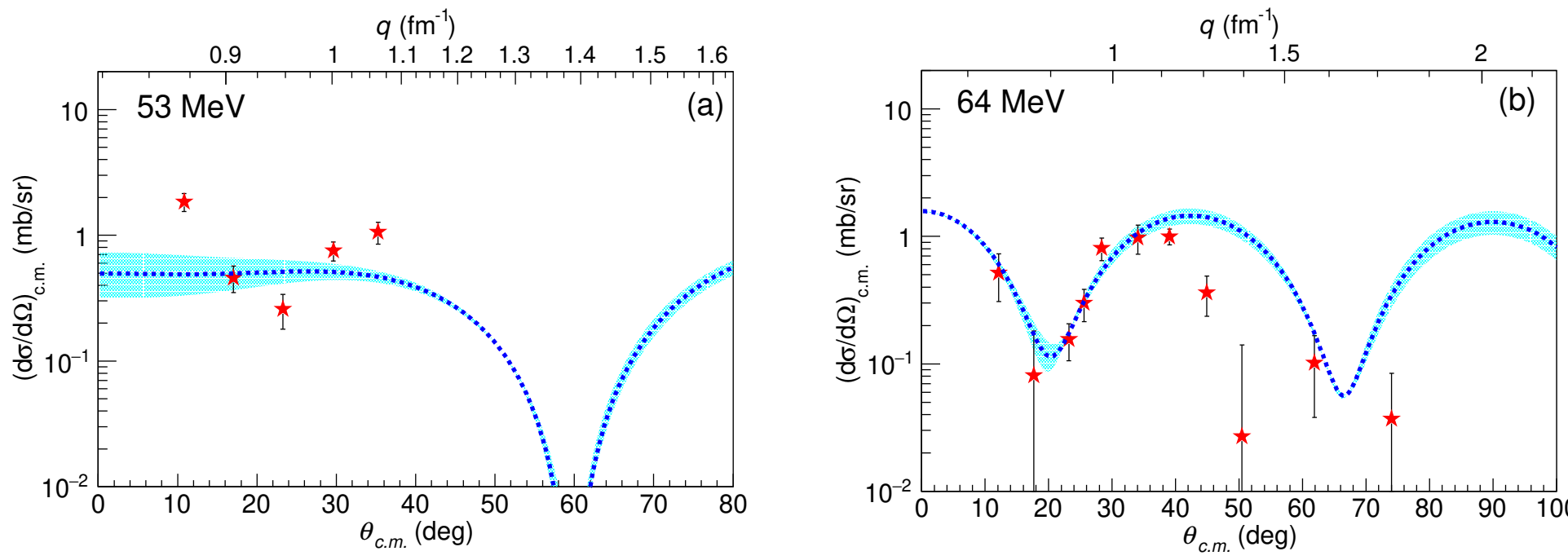

Supplementary Fig. 6 Inelastic scattering angular distributions and uncertainties in the reaction modeling. Experimental data (red stars) for the 0_2^+ resonance at 53 MeV (a) and 64 MeV [41] (b) incident beam energy are compared with the theoretical reaction cross sections using the chiral effective field theory densities from Ref. [10], denoted with the blue dashed line (presented also in Fig. 3 of the main article). The error bars in panel (a) correspond to the combination of uncertainties coming from the solid angle and detection efficiency estimation, the uncertainties in the beam current and the target thickness determination, the statistical error and the background subtraction. The error bars in panel (b) were taken from Ref. [41]. The cyan blue band accounts for the uncertainties in the reaction modeling, common to all calculations but shown here only for the effective field theory densities of Ref. [10].

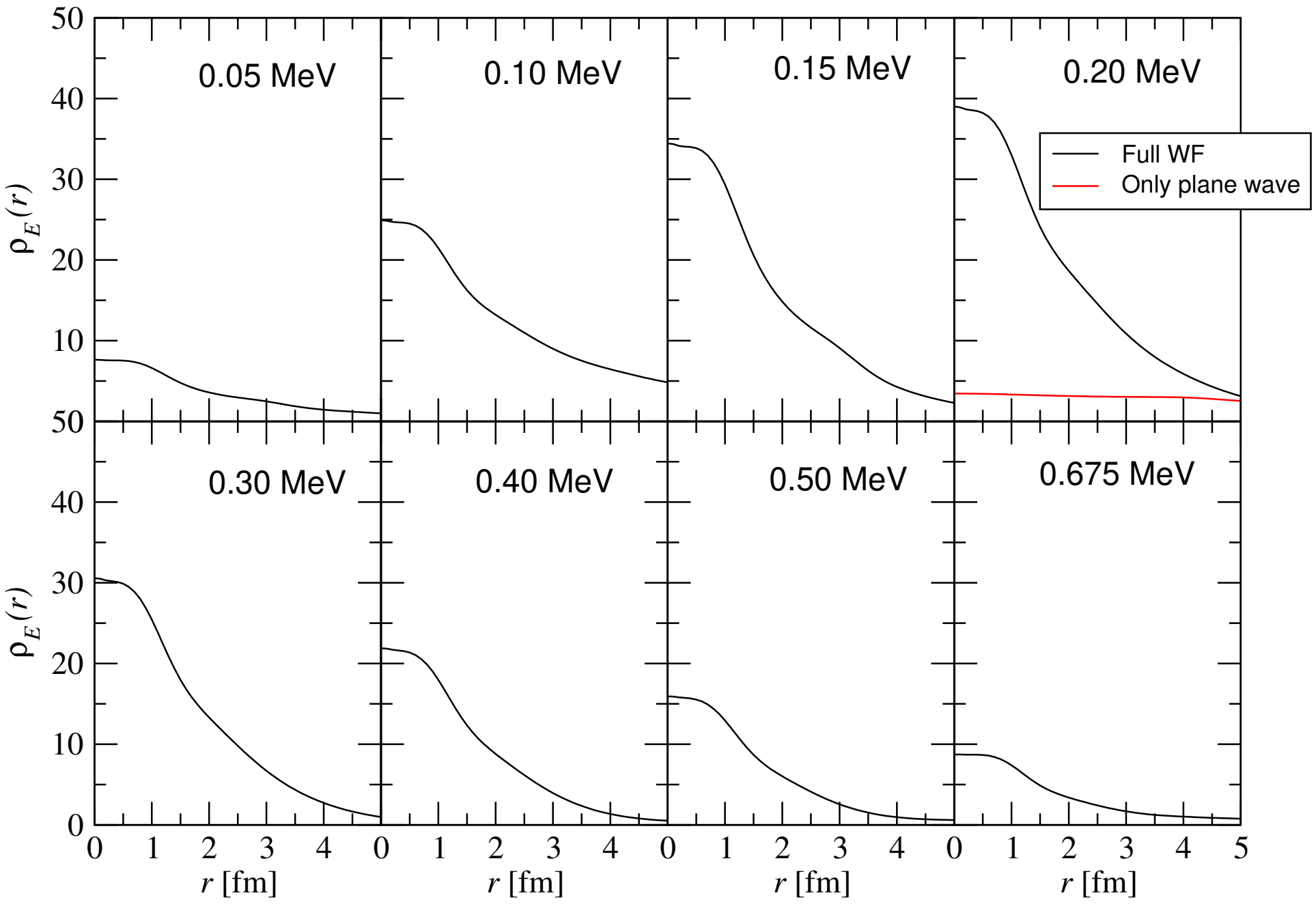

Supplementary Fig. 7 $\rho_E(r)$ **density as defined in Eq. (15), without applying any rescaling factor**. The black and red solid lines represent results adopting the full and the plane wave-functions, respectively (see Supplementary Methods 1.2.1). As $E \to 0.20$ MeV, the particle density for $r < 1$ fm rapidly increases, showing the formation of a quasi-bound state. For the present example the parameters of the resonance can be extracted from $\delta(E)$, and are those given in Refs [29, 42].

3 Supplementary Tables

Supplementary Table 1 Total reaction and inelastic scattering cross sections. The total reaction cross section deduced from the imaginary part of the ISI ($\sigma_{\rm tot}^{\rm th}$) is compared with that extracted from our experimental data and fusion reaction calculations ($\sigma_{\rm tot}^{\rm exp}$). An estimation of the experimental and theoretical inelastic scattering cross section is also denoted as $\sigma_{\rm inel}^{\rm exp}$ and $\sigma_{\rm inel}^{\rm th}$, respectively.

	cross section	angular range
$\sigma_{\rm tot}^{\rm exp}$	370 ± 70 mb	Full
$\sigma_{\rm tot}^{\rm th}$	370 ± 90 mb	Full
$\sigma_{\rm inel}^{\rm exp}$	1.35 ± 0.25 mb	$7.7^\circ \leq \theta_{\rm c.m.} \leq 38.4^\circ$
$\sigma_{\rm inel}^{\rm th}$	1.64 ± 0.28 mb	$7.7^\circ \leq \theta_{\rm c.m.} \leq 38.4^\circ$
$\sigma_{\rm inel}^{\rm th}$	9.5 ± 1.6 mb	Full

Supplementary Table 2 N_{R1} and N_{I1} values. N_{R1} and N_{I1} as a function of l from the fit of the experimental S matrix (elastic scattering) for $E_{\rm Lab} = 53.4$ MeV and $E_{\rm Lab} = 63.9$ MeV.

l	N_{R1}	N_{I1}
0	0.974	0.066
2	0.933	0.105
4	0.948	0.091
6	1.384	0.096

Supplementary References

[1] Satchler, G. R. *Direct Nuclear Reactions* (Oxford University Press, Oxford, 1983).

[2] Chamon, L., Carlson, B. & Gasques, L. São Paulo potential version 2 (SPP2) and Brazilian nuclear potential (BNP). *Computer Physics Communications* **267**, 108061 (2021). URL https://www.sciencedirect.com/science/article/pii/S0010465521001739.

[3] Chamon, L. C., Gasques, L. R. & Carlson, B. V. Approximate treatment of relativistic effects in the low-energy $\alpha + \alpha$ scattering. *Phys. Rev. C* **84**, 044607 (2011). URL https://link.aps.org/doi/10.1103/PhysRevC.84.044607.

[4] De Vries, H., De Jager, C. & De Vries, C. Nuclear charge-density-distribution parameters from elastic electron scattering. *Atomic Data and Nuclear Data Tables* **36**, 495–536 (1987). URL https://www.sciencedirect.com/science/article/pii/0092640X87900131.

[5] Satchler, G. & Love, W. Folding model potentials from realistic interactions for heavy-ion scattering. *Physics Reports* **55**, 183–254 (1979). URL https://www.sciencedirect.com/science/article/pii/0370157379900814.

[6] Takigawa, N., Rumin, T. & Ihara, N. Coulomb interaction between spherical and deformed nuclei. *Phys. Rev. C* **61**, 044607 (2000). URL https://link.aps.org/doi/10.1103/PhysRevC.61.044607.

[7] Chamon, L. C. & Morales-Botero, D. F. Folding-type coupling potentials in the context of the generalized rotation–vibration model. *Journal of Physics G: Nuclear and Particle Physics* **45**, 035104 (2018). URL https://doi.org/10.1088/1361-6471/aaa8b5.

[8] Hiyama, E., Gibson, B. F. & Kamimura, M. Four-body calculation of the first excited state of ^{4}He using a realistic nn interaction: $^4\mathrm{He}(e, e^{'})^4\mathrm{He}(0_2^+)$ and the monopole sum rule. *Phys. Rev. C* **70**, 031001 (2004). URL https://link.aps.org/doi/10.1103/PhysRevC.70.031001.

[9] Bacca, S., Barnea, N., Leidemann, W. & Orlandini, G. Examination of the first excited state of ^{4}He as a potential breathing mode. *Phys. Rev. C* **91**, 024303 (2015). URL https://link.aps.org/doi/10.1103/PhysRevC.91.024303.

[10] Viviani, M., Kievsky, A., Marcucci, L. E. & Girlanda, L. Study of the alpha-particle monopole transition form factor. *Few Body Systems* **65**, 74 (2024). URL https://doi.org/10.1007/s00601-024-01940-2.

[11] Meißner, U.-G., Shen, S., Elhatisari, S. & Lee, D. Ab initio calculation of the alpha-particle monopole transition form factor. *Phys. Rev. Lett.* **132**, 062501 (2024). URL https://link.aps.org/doi/10.1103/PhysRevLett.132.062501.

[12] Russell, J. L., Phillips, G. C. & Reich, C. W. Scattering of alpha particles from helium. *Phys. Rev.* **104**, 135–142 (1956). URL https://link.aps.org/doi/10.1103/PhysRev.104.135.

[13] Heydenburg, N. P. & Temmer, G. M. Alpha-alpha scattering at low energies. *Phys. Rev.* **104**, 123–134 (1956). URL https://link.aps.org/doi/10.1103/PhysRev.104.123.

[14] Nilson, R., Kerman, R. O., Briggs, G. R. & Jentschke, W. Alpha-alpha particle scattering in the energy range 12.3 to 22.9 MeV. *Phys. Rev.* **104**, 1673–1680 (1956). URL https://link.aps.org/doi/10.1103/PhysRev.104.1673.

[15] Bacher, A. D. *et al.* Observation of high-lying levels in ^{8}Be from $\alpha - \alpha$ elastic scattering. *Phys. Rev. Lett.* **29**, 1331–1333 (1972). URL https://link.aps.org/doi/10.1103/PhysRevLett.29.1331.

[16] Tombrello, T. A. & Senhouse, L. S. Elastic scattering of alpha particles from helium. *Phys. Rev.* **129**, 2252–2258 (1963). URL https://link.aps.org/doi/10.1103/PhysRev.129.2252.

[17] Chien, W. S. & Brown, R. E. Study of the $\alpha + \alpha$ system below 15 MeV (c.m.). *Phys. Rev. C* **10**, 1767–1784 (1974). URL https://link.aps.org/doi/10.1103/PhysRevC.10.1767.

[18] Chamon, L. C., Carlson, B. V. & Gasques, L. R. $\alpha + \alpha$ scattering reexamined in the context of the são paulo potential. *Phys. Rev. C* **83**, 034617 (2011). URL https://link.aps.org/doi/10.1103/PhysRevC.83.034617.

[19] Bredin, D. J. *et al.* The scattering of alpha particles by helium. *Proceedings of the Royal Society of London. Series A, Mathematical and Physical Sciences* **251**, 143–155 (1959). URL http://www.jstor.org/stable/100757.

[20] Darriulat, P. *Contribution à l'étude de l'interaction $\alpha - \alpha$*. Ph.D. thesis, University of Paris (1965).

[21] Darriulat, P., Igo, G., Pugh, H. G. & Holmgren, H. D. Elastic scattering of alpha particles by helium between 53 and 120 MeV. *Phys. Rev.* **137**, B315–B325 (1965). URL https://link.aps.org/doi/10.1103/PhysRev.137.B315.

[22] Kamada, H. *et al.* Benchmark test calculation of a four-nucleon bound state. *Phys. Rev. C* **64**, 044001 (2001).

[23] Bacca, S., Barnea, N., Leidemann, W. & Orlandini, G. Isoscalar monopole resonance of the alpha particle: A prism to nuclear hamiltonians. *Phys. Rev. Lett.* **110**, 042503 (2013). URL https://link.aps.org/doi/10.1103/PhysRevLett.110.042503.

[24] Kegel, S. *et al.* Measurement of the α-particle monopole transition form factor challenges theory: A low-energy puzzle for nuclear forces? *Phys. Rev. Lett.* **130**, 152502 (2023). URL https://link.aps.org/doi/10.1103/PhysRevLett.130.152502.

[25] Michel, N., Nazarewicz, W. & Płoszajczak, M. Description of the proton-decaying 0_2^+ resonance of the α particle. *Phys. Rev. Lett.* **131**, 242502 (2023). URL https://link.aps.org/doi/10.1103/PhysRevLett.131.242502.

[26] Yin, P. *et al.* α-particle monopole form factors within the ab initio no-core shell model. *Phys. Rev. C* **112**, L031303 (2025). URL https://link.aps.org/doi/10.1103/1pxs-xjtl.

[27] Tilley, D., Weller, H. & Hale, G. Energy levels of light nuclei A = 4. *Nuclear Physics A* **541**, 1–104 (1992).

[28] Michel, N., Nazarewicz, W. & Płoszajczak, M. Erratum: Description of the proton-decaying 0_2^+ resonance of the α particle [phys. rev. lett. 131, 242502 (2023)]. *Phys. Rev. Lett.* **133**, 239901 (2024). URL https://link.aps.org/doi/10.1103/PhysRevLett.133.239901.

[29] Duerinck, P.-Y. *et al.* Excited state of the α particle: A benchmark study. *Phys. Rev. C* **112**, 044001 (2025). URL https://link.aps.org/doi/10.1103/jcd4-8jh8.

[30] Platter, L., Hammer, H.-W. & Meißner, U.-G. Four-boson system with short-range interactions. *Phys. Rev. A* **70**, 052101 (2004). URL https://link.aps.org/doi/10.1103/PhysRevA.70.052101.

[31] Entem, D. R. & Machleidt, R. Accurate charge-dependent nucleon-nucleon potential at fourth order of chiral perturbation theory. *Phys. Rev. C* **68**, 041001 (2003). URL https://link.aps.org/doi/10.1103/PhysRevC.68.041001.

[32] Machleidt, R. & Entem, D. R. Chiral effective field theory and nuclear forces. *Phys. Rept.* **503**, 1–75 (2011).

[33] Epelbaum, E. *et al.* Three nucleon forces from chiral effective field theory. *Phys. Rev. C* **66**, 064001 (2002).

[34] Navrátil, P. Local three-nucleon interaction from chiral effective field theory. *Few-Body Systems* **41**, 117 (2007). URL https://doi.org/10.1007/s00601-007-0193-3.

[35] Baroni, A. *et al.* Local chiral interactions, the tritium Gamow-Teller matrix element, and the three-nucleon contact term. *Phys. Rev. C* **98**, 044003 (2018).

[36] Efros, V. D. Application of the hyperspherical basis in the theory of few-nucleon systems. *Soviet Journal of Nuclear Physics* **29**, 526 (1979). Originally published in Yadernaya Fizika 29, 1055 (1979).

[37] Barnea, N., Leidemann, W. & Orlandini, G. State dependent effective interaction for the hyperspherical formalism. *Phys. Rev. C* **61**, 054001 (2000). URL https://link.aps.org/doi/10.1103/PhysRevC.61.054001.

[38] Barnea, N., Leidemann, W. & Orlandini, G. State-dependent effective interaction for the hyperspherical formalism with noncentral forces. *Nuclear Physics A* **693**, 565 – 578 (2001). URL http://www.sciencedirect.com/science/article/pii/S0375947401007941.

[39] Kievsky, A., Rosati, S., Viviani, M., Marcucci, L. & Girlanda, L. A High-precision variational approach to three- and four-nucleon bound and zero-energy scattering states. *J. Phys. G* **35**, 063101 (2008).

[40] Marcucci, L. E. *et al.* The hyperspherical harmonics method: A tool for testing and improving nuclear interaction models. *Frontiers in Physics* **8** (2020). URL https://www.frontiersin.org/journals/physics/articles/10.3389/fphy.2020.00069.

[41] Gross, E. E., Hungerford, E. V., Malanify, J. J., Pugh, H. G. & Watson, J. W. Investigation of the reaction ^{4}He (^{4}He, ^{4}He) ^{4}He* at 64 MeV. *Phys. Rev.* **178**, 1584–1590 (1969). URL https://link.aps.org/doi/10.1103/PhysRev.178.1584.

[42] Viviani, M., Girlanda, L., Kievsky, A. & Marcucci, L. E. $n+^3$H, $p+^3$He, $p+^3$H, and $n+^3$He scattering with the hyperspherical harmonic method. *Phys. Rev. C* **102**, 034007 (2020). URL https://link.aps.org/doi/10.1103/PhysRevC.102.034007.
