# Supplementary material for: Probing nuclear interactions à la Rutherford: insights on 4He from α scattering
Source: Nat Commun. 2026 Jun 26;17:8003. doi: 10.1038/s41467-026-74761-y (PMC13451439; doi:10.1038/s41467-026-74761-y)
Supplement: Supplementary file 2 [file 41467_2026_74761_MOESM2_ESM.pdf]

Description of Additional Supplementary Files:

Supplementary Data 1: Inelastic and elastic scattering angular distributions for the beam energy of 53 MeV.
