# Supplementary material for: Probing nuclear interactions à la Rutherford: insights on 4He from α scattering
Source: Nat Commun. 2026 Jun 26;17:8003. doi: 10.1038/s41467-026-74761-y (PMC13451439; doi:10.1038/s41467-026-74761-y)
Supplement: Supplementary file 3 [file 41467_2026_74761_MOESM4_ESM.pdf]

nature portfolio

Peer Review File

Probing nuclear interactions à la Rutherford: Insights on 4He from α scattering

Corresponding Author: Dr Vasileios Soukeras

This file contains all reviewer reports in order by version, followed by all author rebuttals in order by version.

Attachments originally included by the reviewers as part of their assessment can be found at the end of this file.

Version 0:

Reviewer comments:

Reviewer #1

(Remarks to the Author)
This work provides the most precise measurement of the 4He 0_2+ resonance. The authors used a novel coincidence setup that combined the MAGNEX magnetic spectrometer, which detected scattered 4He particles, with the OSCAR solid-state telescope array, which detected 3H or 3He decay fragments. This setup enabled the first-ever exclusive measurement of the alpha + alpha -> alpha + p + 3H and alpha + alpha -> alpha + n + 3He breakup channels. This coincidence technique was key to achieving a clean spectral response with high energy resolution, which allowed for a precise determination of the 0_2+ resonance properties. As current theoretical calculations are not able to reproduce the properties of this resonance well, this points to some remaining challenges for nuclear theory to improve the accuracy of near-threshold resonance calculations.

There are several aspects of this work that need to be improved. My most significant concern is that the treatment of systematic errors is inadequate. The quoted +/-0.01 MeV uncertainties for the resonance energy and width are extremely optimistic. There needs to be a more thorough and detailed analysis that properly incorporates modeling bias and quotes systematic error bars.

I have similar concerns about the systematic errors in the theoretical analysis. The double-folding calculations are a simplified treatment of the reaction, and there is no clear attempt to quantify the uncertainties arising from the input densities or the model itself. This is made clear in Figure 3, where none of the theoretical predictions reproduce the key features of the new 53 MeV experimental data, particularly the pronounced minimum. A "best overall agreement" that still fails to match the data's structure is not sufficient. Once these significant issues regarding the systematic error analysis are fully addressed, I would be happy to review the revised paper.

Reviewer #2

(Remarks to the Author)
The paper by Cappuzzello et al. contains a detailed experimental analysis of 4He by means of the 4He+4He scattering measurements. In particular, the authors accessed the α + α → α + p + 3H and the α + α → α + n + 3He channels. The new unique data are essential for our understanding of the 0+2 resonance in 4He, as it lies close to the 3H+p and 3He+n thresholds. The experimental part of the paper is potentially interesting. Unfortunately, the theoretical discussion is confusing, and the paper is inconclusive.
General comments:
A. How does the work compare to the established literature? Important theoretical works are either not cited or ignored. Examples are:
a. Recent references on ab-initio description of the α + α scattering
• Elhatisari et al., Nature 528, 111 (2015).
• Kravvaris et al., Phys. Lett. B 856, 138930 (2024).
• M. Freer et al., Rev. Mod. Phys. 90, 035004 (2018), review of microscopic clustering in light nuclei.
b. Recent references on the ab-initio coupled-channel approach in the context of the channel coupling phenomena in light nuclei

• Kravvaris et al., Phys. Lett. B 856, 138930 (2024). This work discusses channel-coupling effects in light nuclei such as 12Be and the role of 3N forces in the α + α scattering.
• Michel et al. [19,72]. This work, based on the Gamow shell model in the coupled-channel formulation (CCGSM), pointed out the importance of the 3H+p, 3He+n, and d+d continuum channels for the structure of the 0+ resonance in 4He, and demonstrated quantitatively the role of this channel-coupling effect.
• Mercenne et al., Phys. Rev. C 107, L011603 (2023) and Linares Fernandez et al., Phys. Rev. C 108, 044616 (2023): CCGSM applications to the study of resonances and elastic scattering cross sections in light nuclei.
• Elhatisari et al., arXiv:2507.08495. Ab initio lattice study of neutron–alpha scattering with chiral forces at N3LO.
c. Recent references to the microscopic construction of the optical potential
• Rotureau et al, Phys. Rev. C95, 024315 (2017).
• Idini et al., Phys. Rev. Lett. 123, 092501 (2019)

B. Compared to today's standards (see above), the theoretical analysis is not state-of-the-art. It is based on the couple-channel approach from the 1980s, employing the phenomenological folded potential with an imaginary part.

Specific comments:
Abstract:
1. "Here we report a new study of such 4He resonance by 4He + 4He scattering featuring data of unprecedented sensitivity and state-of-art analyses of the spectral line shape and scattering cross sections."
As discussed in point B, the analysis is not state-of-the-art.
Introduction:
2. "The position Er–typically indicated as the centroid-energy above the Sp threshold–and width Γr were calculated with various theories, see Fig. 1."
Figure 1 is misleading as it lumps together the real-energy models that approximate the spectral function of the 0+2 resonance by a Lorentzian form-factor, and the complex-energy model (Michel et al.), which describes this state as a broad Gamow resonant state. The observed structure shows an asymmetric threshold behavior, modelled in this work by the Fano function. So, unless the consistent shape form factor definition is applied across all models, the collection of theoretical results in Fig. 1 is not meaningful.
Moreover, the result by Michel et al. [19] should not be used as it was later corrected in Ref. [72].
3. "First, the resonance should possibly be addressed with methods capable of dealing with the continuum."
Why "possibly"? It makes no sense to describe a broad threshold state as bound.
4. "Second, the large Γr variation observed exclusively with continuum methods—the only ones capable of computing it—is connected to a strong dependence on the modeling of three-nucleon forces."
This is speculative. In addition to the interaction, the value of Γr is dramatically affected by the theoretical method used, e.g., the choice of non-orthogonal continuum channels, treatment of the Pauli principle, the center-of-mass, etc. As a matter of fact, the proximity of the 0+2 resonance to both proton and neutron decay thresholds will modify the hierarchy of the perturbative corrections in the EFT interaction because there is no reason that the EFT interaction calibrated to phase shifts and properties of light nuclei is capable of describing the near-threshold states in a formalism in which resonances are treated as bound states. Sensitivity of the results on the different variants of the EFT interaction should be studied within the same many-body framework, which is not guaranteed in Bacca et al. [16, 39] and Viviani et al. [29]. In fact, only the CCGSM studies [19,72] attempted to study the effect of different EFT interactions on the monopole transition form factor, though neglecting the 3-body term.
5. As a matter of fact, calculations present stark differences, and none of them reproduces all the three known properties (Er, Γr and FM) of the 0+2 state. The mysterious properties of the first excited state of the α particle and their direct link to basic nuclear Hamiltonians are thus calling for further investigations.

These calculations apply inconsistent definitions of Er and Γr, see Comment 2. It is premature (again!) to invoke mystery.

Section 3.1:
6. A third reaction channel, shown to be important for the description of the excited 0+2 state of 4He [19,72], is 2H+2H. Here, this channel has not been considered. Why?

Section 3.2:

7. The ISI and FSI are built in a microscopic approach by folding the nucleon-nucleon interaction with the 0+1 and 0+2 densities as well as the 0+1 → 0+2 transition density obtained from four different ab-initio calculations, all treating the 0+2 resonance as a bound state.

This reaction approach is not microscopic, see Comment 1. How can a broad threshold state be treated as bound in reaction calculations? Aren't the Authors contradicting themselves?

8. Figure 3. If the experimental data (red symbols) are indeed of unprecedented sensitivity (abstract), one must conclude that all theoretical models employed fail, as the experimental trend is not explained. On the other hand, there is a fair agreement between the theoretical models and the old 64 MeV data from 1969 [21]. Objectively speaking, this result is inconclusive.

Section 4:

9. … we conclude that the challenge of describing the 0+2 resonance can be addressed within the current framework of

nuclear Hamiltonians, without requiring modifications or extensions.

This conclusion is premature; see Comment 4 and notice that the resonance energy, width, and diffraction pattern are not reproduced. Also, the usage of “the current framework of nuclear Hamiltonians” is misleading, as the coupled-channel reaction calculations in this paper are based on the schematic nuclear potential, and the ab-initio input (nucleonic densities) is not discriminating.

10. Indeed, establishing a transparent link between the measured cross section in an open quantum system, such as alpha-alpha scattering, and the resonance in one of its subsystems, remains an unresolved problem in quantum mechanics. Authors seem to have overlooked the efforts to reconcile the shell model with nuclear reactions in both the real-energy and complex-energy formulations of the continuum shell model, where the relation between cross-section and poles of the scattering matrix has been extensively discussed.

Extended Data:
11. How do the theoretical densities presented in Figs. 6(a) and (b) compare to experimental data from electron scattering?.

Section 5.2.2:
12. Ideally, both the interference with non-resonant continuum and the threshold effect should be included in the resonance line shape analysis. However, to our knowledge such a theoretical framework is not available to data and approximate schemes are needed.

Both effects find a natural description in the continuum shell model.

Section 7.1:
13. The coupled-channel analysis is based on the folded-potential approach with the BNP nuclear potential of Ref. [65]. It is a reasonable phenomenological tool, used previously to describe the α + α scattering. But it is not state-of-the-art, and it has little to do with ab-initio coupled-channel theory, see the recent references mentioned above. As seen in Fig. 6, the model dependence of nucleonic densities is minor. How can the experimental results obtained in this work be thus used to discriminate between ab-initio models?

To call the reaction theory analysis in this paper microscopic is an overstatement. The calculation is using the optical potential to determine normalization factors multiplying nuclear potentials (eq. 4 in Sec. 7.1) in the diagonal part of ISI and FSI interactions. The nuclear potentials (eq. 5) are calculated by double-folding the phenomenological BNP interaction. The assumption that the matter density is twice the charge density is reasonable for the ground state. Still, for the 0+2 state, the proximity of the nearby reaction thresholds may change this relation. One may go on and on listing conceptual limitations of the reaction calculations in this paper.

One may consider the present calculation adequate in view of the problems with determining microscopically the ab initio optical potentials. However, it is still a long way to go.

Section 7.2:

14. The historical account is fair but misleading regarding Michel’s work [72]. The Authors complain that Ref. [19,72] neglected three-body forces. We first note that 3N forces were not considered in the reaction calculations of Cappuzzello et al. Second, one cannot make a blanket statement about the impact of 3N forces without reference to the NN channel and the underlying Hilbert space. See, e.g., very relevant Fig. 3 and related discussion of Kravvaris et al. on the α–α scattering phase shifts, in which the authors conclude that the choice of 3N-force regulator plays a significant role in determining the behavior of the α–α scattering. Interestingly, their results based on the NN chiral interaction are very close to those obtained with NN+NNlnl, which describe the scattering data well. Also, the quoted “optimal resonance energy” of Ref. [72] is in fact the real energy of the Gamow pole, not the real-energy fit to the Fano form factor. As stated above, these two definitions should not be confused.

15. The normalization of the continuum density discussed below Eq. (16) is misleading as it assumes that the stationary real-energy approach is the correct way of describing broad decaying states.

16. In the range of energies probed in our experiment, the S-wave p + 3H phase shift
δ(E) has a typical resonance behavior, namely a sharp increase reaching the value of
90 deg. In such a case, ΨE tends to have the form of a “quasi-bound” state, namely to
be very large in the region where the four nucleons are close to each other, much larger than ΨA.

Such a behavior is not obvious in Fig. 7.

Version 1:

Reviewer comments:

Reviewer #1

(Remarks to the Author)
The authors have addressed my concerns adequately. I am happy to recommend publication.

Reviewer #2

(Remarks to the Author)
The manuscript has been much improved in the process. In particular several overstatements have been toned down. The additional Fig. 10 is helpful. The only comment I have is about the caption of Fig. 1. There are still remnants of the previous caption "(right)", "while,". Also, the proton-emission threshold Sp = 19.813 MeV is experimental, not "theoretical".

Open Access This Peer Review File is licensed under a Creative Commons Attribution 4.0 International License, which permits use, sharing, adaptation, distribution and reproduction in any medium or format, as long as you give appropriate credit to the original author(s) and the source, provide a link to the Creative Commons license, and indicate if changes were made.
In cases where reviewers are anonymous, credit should be given to 'Anonymous Referee' and the source.
The images or other third party material in this Peer Review File are included in the article's Creative Commons license, unless indicated otherwise in a credit line to the material. If material is not included in the article's Creative Commons license and your intended use is not permitted by statutory regulation or exceeds the permitted use, you will need to obtain permission directly from the copyright holder.
To view a copy of this license, visit https://creativecommons.org/licenses/by/4.0/

Response to reviewers comments

We thank both referees for carefully reading the article as well as for the constructive criticisms provided. Their comments have helped us to significantly improve the manuscript. Below, we address each point in turn, first quoting the referee's comment (in black) and then providing our response labeled by "Answer" (in blue). We have also submitted the revised manuscript, with all changes clearly highlighted in color.

Reviewer #1

This work provides the most precise measurement of the ^{4}He 0_2^+ resonance. The authors used a novel coincidence setup that combined the MAGNEX magnetic spectrometer, which detected scattered ^{4}He particles, with the OSCAR solid-state telescope array, which detected ^{3}H or ^{3}He decay fragments. This setup enabled the first-ever exclusive measurement of the alpha + alpha → alpha + p + ^{3}H and alpha + alpha → alpha + n + ^{3}He breakup channels. This coincidence technique was key to achieving a clean spectral response with high energy resolution, which allowed for a precise determination of the 0_2^+ resonance properties. As current theoretical calculations are not able to reproduce the properties of this resonance well, this points to some remaining challenges for nuclear theory to improve the accuracy of near-threshold resonance calculations.

There are several aspects of this work that need to be improved. My most significant concern is that the treatment of systematic errors is inadequate. The quoted ±0.01 MeV uncertainties for the resonance energy and width are extremely optimistic. There needs to be a more thorough and detailed analysis that properly incorporates modeling bias and quotes systematic error bars.

Answer: We thank the referee for the constructive criticism. The comments have helped us improve the manuscript significantly. The uncertainties were revised such as to include a more comprehensive analysis of the systematic uncertainties. The revised errors include the uncertainty in the energy threshold effect on the line shape model, the deconvolution of the spectrum in the three components (see Fig. 2), the contribution of the finite bin size, the accuracies in energy or/and angle of the delivered beam, the spectrometer and the ray-reconstruction procedure as well as the variation of the experimental resolution as a

function of angle (see Section 5.2.1). The new uncertainties implementing the Fano analysis are ±0.02 MeV and ±0.04 MeV for the resonance energy and width, respectively. For the sake of a more direct comparison with traditional analyses found in the literature we also evaluate the centroid, the width and revive their uncertainties assuming a Lorentzian shape. The values were appropriately corrected in Fig. 1 and Section 3.1, while, an explanation was included in Section 5.2.2. We thank the referee for pointing out that issue.

I have similar concerns about the systematic errors in the theoretical analysis. The double-folding calculations are a simplified treatment of the reaction, and there is no clear attempt to quantify the uncertainties arising from the input densities or the model itself. This is made clear in Figure 3, where none of the theoretical predictions reproduce the key features of the new 53 MeV experimental data, particularly the pronounced minimum. A "best overall agreement" that still fails to match the data's structure is not sufficient. Once these significant issues regarding the systematic error analysis are fully addressed, I would be happy to review the revised paper.

Answer: We agree with the referee that the double-folding calculation represents a simplified treatment of the reaction, and we have clarified this point in the revised manuscript. Nevertheless, we would like to emphasize that we have made a concrete effort to quantify uncertainties within the limits of the current theoretical framework. Regarding the uncertainties associated with the input densities, Fig. 3 includes two bands: one reflecting the spread between the two chiral EFT densities, and one capturing the variation between the two phenomenological potentials. These bands can be interpreted as an estimate of the uncertainty on the input densities. We now made this more explicit on pages 7-8. Concerning the reaction model itself, we address it in Section 5.4, in Fig. 8 and 9. For example, in Fig. 9 the cyan band represents the uncertainty arising from the reaction modeling. This latter has been evaluated by varying the initial and final state interaction parameters within the constraints provided by elastic scattering and total reaction cross sections and probing the same methodology also to the data at 64 MeV. We find that the overall contribution from reaction modeling is comparable across all calculations; however, for clarity, it is shown only for the EFT densities of Ref. [29].

Going beyond the present model, while simultaneously employing the same densities used in electron-scattering analyses, is not straightforward and, presently, not feasible to our knowledge. We nevertheless believe that the new data, together with the theoretical comparison

presented here, can serve as a strong motivation to advance theoretical descriptions beyond the current state of the art, which remains, for now, the only viable approach.

Answer to Reviewer #2

We thank the referee for the constructive criticisms provided on the theory. These comments have helped us improve the manuscript significantly, and we believe the revised version is now stronger.

A. How does the work compare to the established literature? Important theoretical works are either not cited or ignored. Examples are:

a. Recent references on ab-initio description of the $\alpha + \alpha$ scattering

- Elhatisari et al., Nature 528, 111 (2015).
- Kravvaris et al., Phys. Lett. B 856, 138930 (2024).
- M. Freer et al., Rev. Mod. Phys. 90, 035004 (2018), review of microscopic clustering in light nuclei.

b. Recent references on the ab-initio coupled-channel approach in the context of the channel coupling phenomena in light nuclei

- Kravvaris et al., Phys. Lett. B 856, 138930 (2024). This work discusses channel-coupling effects in light nuclei such as ^{12}Be and the role of 3N forces in the $\alpha + \alpha$ scattering.
- Michel et al. [19,72]. This work, based on the Gamow shell model in the coupled-channel formulation (CCGSM), pointed out the importance of the ^{3}H+p, ^{3}He+n, and d+d continuum channels for the structure of the 0+ resonance in ^{4}He, and demonstrated quantitatively the role of this channel-coupling effect.
- Mercenne et al., Phys. Rev. C 107, L011603 (2023) and Linares Fernandez et al., Phys. Rev. C 108, 044616 (2023): CCGSM applications to the study of resonances and elastic scattering cross sections in light nuclei.
- Elhatisari et al., arXiv:2507.08495. Ab initio lattice study of neutron–alpha scattering with chiral forces at N3LO.

c. Recent references to the microscopic construction of the optical potential

- Rotureau et al, Phys. Rev. C95, 024315 (2017).
- Idini et al., Phys. Rev. Lett. 123, 092501 (2019)

Answer: We thank the referee for bringing these references to our attention. While they are not directly related to the inelastic $\alpha-\alpha$ reaction cross section, we agree that mentioning (most of) them in the introduction appropriately acknowledges the broader efforts of the community and strengthens the context of our work. We therefore added a paragraph in the introduction.

B. Compared to today's standards (see above), the theoretical analysis is not state-of-the art. It is based on the couple-channel approach from the 1980s, employing the phenomenological folded potential with an imaginary part.

Answer: After carefully reviewing all the papers suggested by the referee, we confirm our original assessment: at present, no ab initio reaction framework has addressed inelastic α–α scattering as an eight-body problem in the continuum. The approach we employ here is rooted in a well-established reaction theory, extensively tested since the 1980s, and provides a robust foundation for investigating the unresolved issues identified in the Kegel et al. paper and subsequent works. Our contribution introduces a new probe—inelastic α–α scattering—and, although the folded potential remains phenomenological, we combine it with state-of-the-art densities obtained from ab initio nuclear structure calculations. This represents a significant advance over the traditional implementations of the theory, which relied exclusively on phenomenological or parametrized densities. In contrast, we use the same high-precision densities that have been used to study electron–scattering. These densities result from large-scale many-body calculations and, in principle, encode implicitly all relevant cluster configurations of the α particle that may appear in the final scattering state. We therefore believe that our approach constitutes a meaningful and necessary step forward in confronting this problem with the best microscopic input currently available.
We have rephrased the text accordingly.

Specific comments:

Abstract:

1. *"Here we report a new study of such ^4He resonance by ^4He + ^4He scattering featuring data of unprecedented sensitivity and state-of-art analyses of the spectral line shape and scattering cross sections."*

As discussed in point B, the analysis is not state-of-the-art.

Answer: We have rephrased the abstract to address this point and better describe our method. The term "state-of-the-art" is now used exclusively to describe the ab-initio densities.

Introduction:

2. *"The position Er–typically indicated as the centroid-energy above the Sp threshold–and width Γ_r were calculated with various theories, see Fig. 1."*

Figure 1 is misleading as it lumps together the real-energy models that approximate the spectral function of the 0_2^+ resonance by a Lorentzian form-factor, and the complex-energy model (Michel et al.), which describes this state as a broad Gamow resonant state. The observed structure shows an asymmetric threshold behaviour, modelled in this work by the Fano function. So, unless the consistent shape form factor definition is applied across all models, the collection of theoretical results in Fig. 1 is not meaningful.

Moreover, the result by Michel et al. [19] should not be used as it was later corrected in Ref. [72].

Answer: Although the left panel of Fig. 1 originally included theoretical results compiled from the literature, which had its own value, we have removed it to avoid the potential confusion noted by the referee. Figure 1 now presents only the experimental data. The corresponding paragraph of the Introduction has been rephrased, and the discussion of the theory has been shortened.

3. *"First, the resonance should possibly be addressed with methods capable of dealing with the continuum."*

Why "possibly"? It makes no sense to describe a broad threshold state as bound.

Answer: We have removed this part.

4. *"Second, the large Γ_r variation observed exclusively with continuum methods—the only ones capable of computing it—is connected to a strong dependence on the modelling of three*

nucleon forces."

This is speculative. In addition to the interaction, the value of Γ_r is dramatically affected by the theoretical method used, e.g., the choice of non-orthogonal continuum channels, treatment of the Pauli principle, the centre-of-mass, etc. As a matter of fact, the proximity of the 0+2 resonance to both proton and neutron decay thresholds will modify the hierarchy of the perturbative corrections in the EFT interaction because there is no reason that the EFT interaction calibrated to phase shifts and properties of light nuclei is capable of describing the near-threshold states in a formalism in which resonances are treated as bound states. Sensitivity of the results on the different variants of the EFT interaction should be studied within the same many-body framework, which is not guaranteed in Bacca et al. [16, 39] and Viviani et al. [29]. In fact, only the CCGSM studies [19,72] attempted to study the effect of different EFT interactions on the monopole transition form factor, though neglecting the 3-body term.

Answer: As pointed out above, we have completely removed the theory panel in Fig. 1 and have shortened the discussion, where we say that the values of (E_r, Γ_r) depend on the method and on the force.

5. *As a matter of fact, calculations present stark differences, and none of them reproduces all the three known properties (E_r, Γ_r and FM) of the 0_2^+ state. The mysterious properties of the first excited state of the α particle and their direct link to basic nuclear Hamiltonians are thus calling for further investigations.*

These calculations apply inconsistent definitions of Er and Γ_r, see Comment 2. It is premature (again!) to invoke mystery.

Answer: We have rephrased avoiding the use of the term mysterious.

Section 3.1:

6. A third reaction channel, shown to be important for the description of the excited 0_2^+ state of ^{4}He [19,72], is ^{2}H+^{2}H. Here, this channel has not been considered. Why?

Answer: Our theoretical description is based on a two–level model consisting of the ground state 0_1^+ and the excited state 0_2^+ treated as a bound state. We specify this in the revised manuscript. The 0_2^+ state is not explicitly decomposed into individual reaction channels, but is obtained from ab-initio calculations. Explicitly decomposing the 0_2^+ state

into separate channels would require more sophisticated approaches, such as Continuum Discretized Coupled Channel calculations, where the inclusion of ab-initio densities would not be practical and further modeling would be required. Although the ^{2}H + ^{2}H channel mentioned by the referee is, of course, included in Refs. [19,72] (Refs. [19,78] of the new manuscript), it is treated there within a four–body calculation. For the present case of $\alpha + \alpha$ scattering, a corresponding inclusion in a eight–body calculation would be required, which represents a significantly more demanding problem and, to the best of our knowledge, has not yet been explored in the literature. For these reasons, we remain confident that our two-level approach is meaningful as a first practical attempt to analyze this reaction.

Section 3.2:

7. *The ISI and FSI are built in a microscopic approach by folding the nucleon-nucleon interaction with the 0_1^+ and 0_2^+ densities as well as the $0_1^+ \to 0_2^+$ transition density obtained from four different ab-initio calculations, all treating the 0_2^+ resonance as a bound state.*

This reaction approach is not microscopic, see Comment 1. How can a broad threshold state be treated as bound in reaction calculations? Aren't the Authors contradicting themselves?

Answer: Although the 0_2^+ is clearly not a bound state, we show in Fig. 6d that a calculation of the density of the 0_2^+ resonance approximated as a bound state is not very different to a calculation including the continuum, especially in the range probed by our $\alpha + \alpha$ scattering experiment below 3 fm. Therefore, again, we remain confident that our approach is solid.
With respect to the question of whether our approach can be considered microscopic, we address this in Point 13.

8. Figure 3. If the experimental data (red symbols) are indeed of unprecedented sensitivity (abstract), one must conclude that all theoretical models employed fail, as the experimental trend is not explained. On the other hand, there is a fair agreement between the theoretical models and the old 64 MeV data from 1969 [21]. Objectively speaking, this result is inconclusive.

Answer: We do not agree that the appropriate conclusion is that all models fail, and therefore this paper is inconclusive. As shown in Fig. 3, the chiral interactions (blue curves)

provide a noticeably better description of the data than the simpler interactions (black curves), indicating that the present measurements can indeed discriminate between models. While we concur that this is more pronounced at 64 MeV than at 53 MeV, it remains evident that at 53 MeV the simpler models (black) do not even reproduce the correct order of magnitude.

Section 4:

9. ... *we conclude that the challenge of describing the* 0_2^+ *resonance can be addressed within the current framework of nuclear Hamiltonians, without requiring modifications or extensions.*

This conclusion is premature; see Comment 4 and notice that the resonance energy, width, and diffraction pattern are not reproduced. Also, the usage of "the current framework of nuclear Hamiltonians" is misleading, as the coupled-channel reaction calculations in this paper are based on the schematic nuclear potential, and the ab-initio input (nucleonic densities) is not discriminating.

Answer: We now agree with the referee that these conclusions were a bit premature and perhaps too strong. In the revised manuscript, we have substantially rephrased the conclusions.

10. *Indeed, establishing a transparent link between the measured cross section in an open quantum system, such as alpha-alpha scattering, and the resonance in one of its subsystems, remains an unresolved problem in quantum mechanics.*

Authors seem to have overlooked the efforts to reconcile the shell model with nuclear reactions in both the real-energy and complex-energy formulations of the continuum shell model, where the relation between cross-section and poles of the scattering matrix has been extensively discussed.

Answer: We have now included citations to the efforts aimed at reconciling the shell model with reaction theory (see point A). However, to the best of our knowledge, none of these approaches is currently capable of providing a truly *ab initio* description of the α–α inelastic scattering data presented in this work. The reaction under consideration involves a breakup process with two clusters in the initial state and three clusters in the final state, all interacting through the Coulomb force. Even the simplest breakup channel, $\alpha + p + {}^3\text{H}$,

remains beyond the present capabilities of any existing method. We have anyway rephrased the conclusion around the sentence quoted by the referee in this point.

Extended Data:

11. How do the theoretical densities presented in Figs. 6(a) and (b) compare to experimental data from electron scattering?

Answer: The densities we use here are the same as those employed in studies of electron scattering. For the inelastic monopole form factor, only the transition density is relevant. As noted in the cited works, the densities calculated by Hiyama et al., Meißner et al., and Viviani et al. reproduce the electron scattering data accurately, whereas those of Bacca et al. do not. It is important to emphasize, however, that in $\alpha - \alpha$ inelastic scattering the density of the 0_2^+ state plays a significant role, while it is irrelevant for electron scattering. Therefore, in this work we probe new aspects of the densities.

Section 5.2.2:

12. *Ideally, both the interference with non-resonant continuum and the threshold effect should be included in the resonance line shape analysis. However, to our knowledge such a theoretical framework is not available to date and approximate schemes are needed.*

Both effects find a natural description in the continuum shell model.

To the best of our knowledge, the continuum shell model has not yet been applied to an inelastic $\alpha - \alpha$ scattering process.

Section 7.1:

13. The coupled-channel analysis is based on the folded-potential approach with the BNP nuclear potential of Ref. [65]. It is a reasonable phenomenological tool, used previously to describe the $\alpha + \alpha$ scattering. But it is not state-of-the-art, and it has little to do with ab-initio coupled channel theory, see the recent references mentioned above. As seen in Fig. 6, the model dependence of nucleonic densities is minor. How can the experimental results obtained in this work be thus used to discriminate between ab-initio models?

To call the reaction theory analysis in this paper microscopic is an overstatement. The calculation is using the optical potential to determine normalization factors multiplying nuclear potentials (eq. 4 in Sec. 7.1) in the diagonal part of ISI and FSI interactions. The nuclear potentials (eq. 5) are calculated by double-folding the phenomenological BNP

interaction. The assumption that the matter density is twice the charge density is reasonable for the ground state. Still, for the 0_2^+ state, the proximity of the nearby reaction thresholds may change this relation. One may go on and on listing conceptual limitations of the reaction calculations in this paper.

One may consider the present calculation adequate in view of the problems with determining microscopically the ab initio optical potentials. However, it is still a long way to go.

Answer: As the referee notes, the folded-potential approach is indeed a reasonable phenomenological tool. Moreover, it is currently the only practical framework available for analyzing inelastic α–α scattering in the absence of a full ab-initio reaction theory, which to the best of our knowledge has not yet been demonstrated in the literature. While awaiting more complete theoretical developments, which could well come from the coupled-channel shell model, we presently need to proceed with something that is solid and available. Therefore, we have analyzed the new data using the folded potential reaction theory. The novelty is that we used densities obtained from four different ab-initio structure calculations. In this sense, our work goes well beyond what was done in the 1980s.

The referee claims that the model dependence in Fig. 6 is small and one can therefore not discriminate among theories using the new data. This is not true. We find that the $\alpha - \alpha$ inelastic scattering is particularly sensitive to the density of the 0_2^+ state in the range between 2 and 5 fm, where on Fig. 6 one can appreciate some differences despite the logarithmic scale. The difference is then amplified at the level of the cross sections displayed in Fig. 3.

The referee argues that calling this reaction theory "microscopic" is an overstatement. We would like to clarify that, when we wrote the first manuscript, we used the term 'microscopic' not as a synonymous of 'ab-initio'. We considered the present method microscopic because the reaction potential is constructed by folding the BNP nucleon–nucleon interaction with nuclear densities that themselves originate from ab-initio structure calculations where the four-body dynamics is fully resolved. However, to avoid misunderstanding, since we are aware of the limitations of our theoretical approach and we have no intention to hide them, we opted for calling our approach "semi-phenomenological", and have rephrased the revised manuscript accordingly.

Finally, as the referee recognizes "One may consider the present calculation adequate in view of the problems with determining microscopically the ab initio optical potentials. However,

it is still a long way to go". In the revised version, we have explicitly included the point regarding optical potentials in the conclusions.

Section 7.2:

14. The historical account is fair but misleading regarding Michel's work [72]. The Authors complain that Ref. [19,72] neglected three-body forces. We first note that 3N forces were not considered in the reaction calculations of Cappuzzello et al. Second, one cannot make a blanket statement about the impact of 3N forces without reference to the NN channel and the underlying Hilbert space. See, e.g., very relevant Fig. 3 and related discussion of Kravvaris et al. on the α–α scattering phase shifts, in which the authors conclude that the choice of 3N-force regulator plays a significant role in determining the behavior of the α–α scattering. Interestingly, their results based on the NN chiral interaction are very close to those obtained with NN+NN$_{lnl}$, which describe the scattering data well. Also, the quoted "optimal resonance energy" of Ref. [72] is in fact the real energy of the Gamow pole, not the real-energy fit to the Fano form factor. As stated above, these two definitions should not be confused.

Answer: We appreciate the referee points that one cannot make a general statement about the role of three nucleon forces, because their effect is different on different observables. However, while it was found not to be large in Kravvaris et al. paper, $\alpha-\alpha$ elastic scattering (addressed by Kravvaris) and inelastic $\alpha-\alpha$ scattering (addressed by us) could be quite different. We remind the referee that in our calculations, although the BNP potential itself does not include a three-body force, all four ab-initio calculations of the densities do incorporate three-body forces. However, to avoid any potential contention regarding the role of three-body forces, in Sect. 7.2 we have removed the phrase 'nowadays considered state-of-the-art' when referring to the work of Michel et al. We felt that this wording might have been unnecessarily strong and could have contributed to the referee's concern.

Following the referee comment, we realized that the real energy of the Gamow pole should not be confused with the actual centroid energy of the resonance that we have extracted. Indeed, if Michel et al. use the "experimental" value of 0.4 MeV for the centroid resonance to tune their Hamiltonian, we guess that the real energy of the Gamow pole is a different quantity that should not be compared to the experimental centroid energy. Therefore, to avoid misunderstanding, we rephrased the relevant sentence and removed "not close to

experiment" from the text.

15. The normalization of the continuum density discussed below Eq. (16) is misleading as it assumes that the stationary real-energy approach is the correct way of describing broad decaying states.

Answer: Here we disagree with the referee. Clearly (broad) resonant states can be studied by various theoretical methods. We think that real energy methods can give interesting information on resonances. To be specific, we have studied the $0_2^+ \to p + {}^3\mathrm{H}$ scattering state at centre-of-mass energy E. An output of the calculation is the phase shift $\delta(E)$. The variation with the energy of such a quantity shows the typical behavior in case of the presence of a resonant state: a sharp increase, with $\delta(E)$ reaching 90°. From $\delta(E)$, there are well established methods in order to extract the resonance parameters, as the time-delay formalism [Phys. Rev. 118, 349 (1960)], Pade' methods [Phys. A: Math. Theor. 40, 14857 (2007], etc. In particular, looking to the quantity $\rho_E(r)$, as defined in Eq.(16) we clearly see signs of the formation of the resonance as shown in the plot below (in this case we do not apply any rescaling factor to ρ_E).

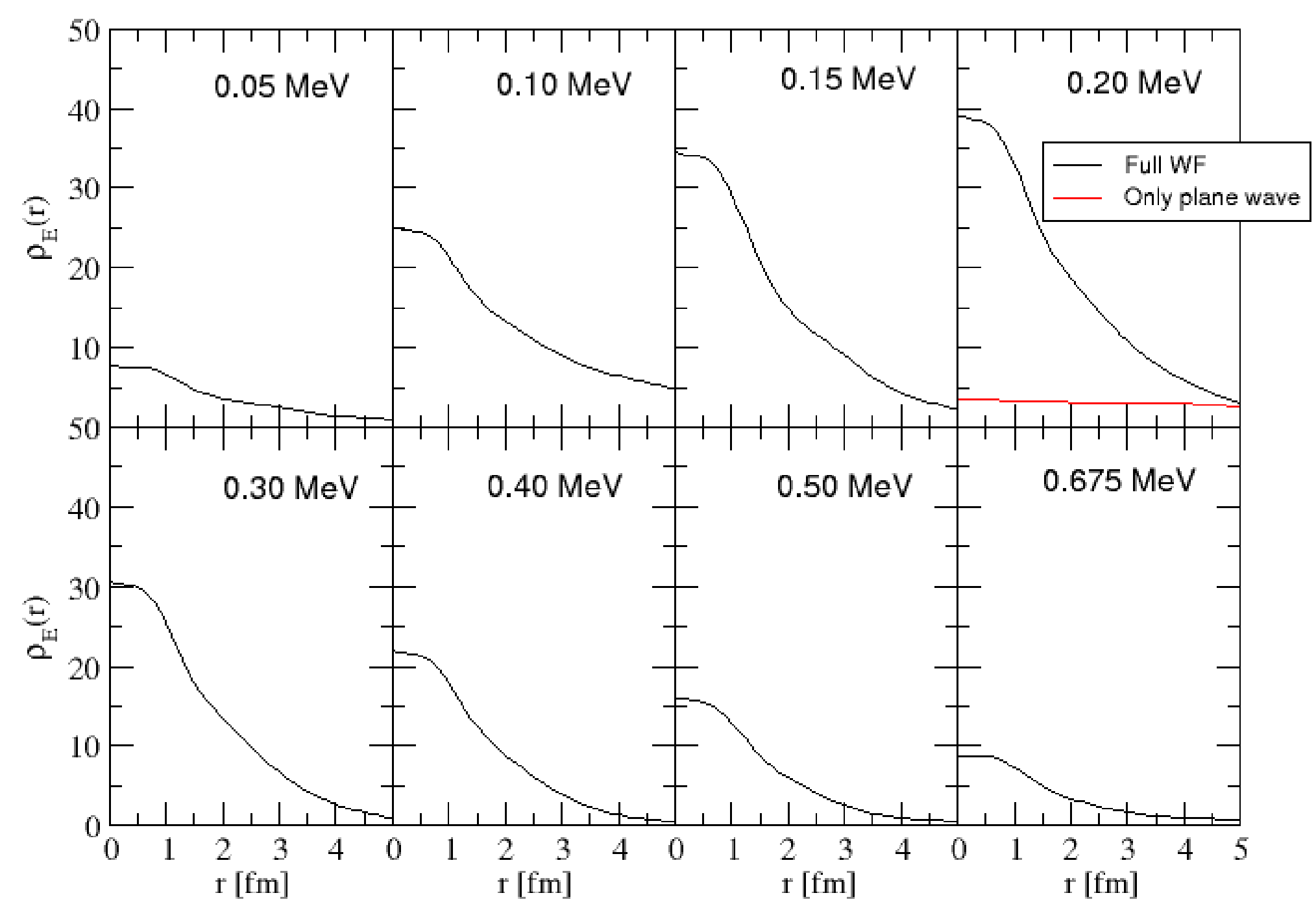

In fact, as $E \to 0.20$ MeV, the particle density for $r < 1$ fm rapidly increases, showing the formation of a quasi-bound state. To be noted that in this specific case the parameters of the resonance can be extracted from $\delta(E)$ and are those given in Refs. [14,17].

16. In the range of energies probed in our experiment, the S-wave p + 3H phase shiftδ(E) has a typical resonance behavior, namely a sharp increase reaching the value of 90 deg. In such a case, ΨE tends to have the form of a "quasi-bound" state, namely to be very large in the region where the four nucleons are close to each other, much larger than ΨA.

Such a behavior is not obvious in Fig. 7.

Answer: Such a behaviour is shown in the above figure. We introduced the figure in "Extended Data Figures" (as Fig. 10) together with a short comment in the last paragraph of Supplementary Information Section 7.2.1.

Summary

The new data probing the ^{3}He+n and ^{3}H+p continuum structure of ^{4}He are interesting and, in principle, important. The theoretical discussion, based on a traditional approach, is sound, but it has limited predictive power. The paper is inconclusive, and some discussion is overstated.

Answer: We agree that some parts of the discussion were overstated, and in the resubmission we have addressed this point thoroughly. As the referee notes, our approach is sound, and we maintain that it provides essentially the maximum predictive power achievable with the currently available theoretical tools for the inelastic ^{4}He+^{4}He scattering. Should a more microscopic treatment become feasible in the near future, it would represent a major breakthrough. We believe that the new data presented in this paper can serve as a strong motivation to advance theory beyond the current state of the art, which remains, at present, the only viable approach.

In addition, the better agreement shown by chiral densities compared to phenomenological ones for the inelastic ^{4}He+^{4}He scattering is a valuable result for the community, since this process is particularly sensitive to the details of the 0_2^+ resonance spatial distribution well beyond the ^{4}He ground state radius, not probed by other processes such as electron scattering.

The paper by Cappuzzello et al. contains a detailed experimental analysis of ^{4}He by means of the ^{4}He+^{4}He scattering measurements. In particular, the authors accessed the α + α → α + p + ^{3}H and the α + α → α + n + ^{3}He channels. The new unique data are essential for our understanding of the 0^+_2 resonance in ^{4}He, as it lies close to the ^{3}H+p and ^{3}He+n thresholds. The experimental part of the paper is potentially interesting. Unfortunately, the theoretical discussion is confusing, and the paper is inconclusive.

General comments:

A. How does the work compare to the established literature? Important theoretical works are either not cited or ignored. Examples are:
   a. Recent references on ab-initio description of the α + α scattering
      - Elhatisari et al., Nature 528, 111 (2015).
      - Kravvaris et al., Phys. Lett. B 856, 138930 (2024).
      - M. Freer et al., Rev. Mod. Phys. 90, 035004 (2018), review of microscopic clustering in light nuclei.
   b. Recent references on the ab-initio coupled-channel approach in the context of the channel coupling phenomena in light nuclei
      - Kravvaris et al., Phys. Lett. B 856, 138930 (2024). This work discusses channel-coupling effects in light nuclei such as ^{12}Be and the role of 3N forces in the α + α scattering.
      - Michel et al. [19,72]. This work, based on the Gamow shell model in the coupled-channel formulation (CCGSM), pointed out the importance of the ^{3}H+p, ^{3}He+n, and d+d continuum channels for the structure of the 0^+ resonance in ^{4}He, and demonstrated quantitatively the role of this channel-coupling effect.
      - Mercenne et al., Phys. Rev. C 107, L011603 (2023) and Linares Fernandez et al., Phys. Rev. C 108, 044616 (2023): CCGSM applications to the study of resonances and elastic scattering cross sections in light nuclei.
      - Elhatisari et al., arXiv:2507.08495. Ab initio lattice study of neutron–alpha scattering with chiral forces at N3LO.
   c. Recent references to the microscopic construction of the optical potential
      - Rotureau et al, Phys. Rev. C95, 024315 (2017).
      - Idini et al., Phys. Rev. Lett. 123, 092501 (2019)

B. Compared to today’s standards (see above), the theoretical analysis is not state-of-the-art. It is based on the couple-channel approach from the 1980s, employing the phenomenological folded potential with an imaginary part.

Specific comments:

Abstract:

1. *"Here we report a new study of such 4He resonance by 4He + 4He scattering featuring data of unprecedented sensitivity and state-of-art analyses of the spectral line shape and scattering cross sections."*

   As discussed in point B, the analysis is not state-of-the-art.

Introduction:

2. *"The position E_r–typically indicated as the centroid-energy above the Sp threshold–and width Γ_r were calculated with various theories, see Fig. 1."*

   Figure 1 is misleading as it lumps together the real-energy models that approximate the spectral function of the 0^+_2 resonance by a Lorentzian form-factor, and the complex-energy model (Michel et al.), which describes this state as a broad Gamow resonant state. The observed structure shows an asymmetric threshold behavior, modelled in this work by the Fano function. So, unless the consistent shape form factor definition is applied across all models, the collection of theoretical results in Fig. 1 is not meaningful.

   Moreover, the result by Michel et al. [19] should not be used as it was later corrected in Ref. [72].

3. *"First, the resonance should possibly be addressed with methods capable of dealing with the continuum."*

   Why "possibly"? It makes no sense to describe a broad threshold state as bound.

4. *"Second, the large Γ_r variation observed exclusively with continuum methods—the only ones capable of computing it—is connected to a strong dependence on the modeling of three-nucleon forces."*

   This is speculative. In addition to the interaction, the value of Γ_r is dramatically affected by the theoretical method used, e.g., the choice of non-orthogonal continuum channels, treatment of the Pauli principle, the center-of-mass, etc. As a matter of fact, the proximity of the 0^+_2 resonance to both proton and neutron decay thresholds will modify the hierarchy of the perturbative corrections in the EFT interaction because there is no reason that the EFT interaction calibrated to phase shifts and properties of light nuclei is capable of describing the near-threshold states in a formalism in which resonances are treated as bound states. Sensitivity of the results on the different variants of the EFT interaction should be studied within the same many-body framework, which is not guaranteed in Bacca et al. [16, 39] and Viviani et al. [29]. In fact, only the CCGSM studies [19,72] attempted to study the effect of different EFT interactions on the monopole transition form factor, though neglecting the 3-body term.

5. *As a matter of fact, calculations present stark differences, and none of them reproduces all the three known properties (E_r, Γ_r and FM) of the* 0^+_2 *state. The mysterious properties of the first excited state of the α particle and their direct link to basic nuclear Hamiltonians are thus calling for further investigations.*

   These calculations apply inconsistent definitions of E_r and Γ_r, see Comment 2. It is premature (again!) to invoke mystery.

Section 3.1:

6. A third reaction channel, shown to be important for the description of the excited 0^+_2 state of ^4He [19,72], is $^2H+^2H$. Here, this channel has not been considered. Why?

Section 3.2:

7. *The ISI and FSI are built in a microscopic approach by folding the nucleon-nucleon interaction with the* 0^+_1 *and* 0^+_2 *densities as well as the* $0^+_1 \rightarrow 0^+_2$ *transition density obtained from four different ab-initio calculations, all treating the* 0^+_2 *resonance as a bound state.*

   This reaction approach is not microscopic, see Comment 1. How can a broad threshold state be treated as bound in reaction calculations? Aren't the Authors contradicting themselves?

8. Figure 3. If the experimental data (red symbols) are indeed of *unprecedented sensitivity* (abstract), one must conclude that all theoretical models employed fail, as the experimental trend is not explained. On the other hand, there is a fair agreement between the theoretical models and the old 64 MeV data from 1969 [21]. Objectively speaking, this result is inconclusive.

Section 4:

9. … *we conclude that the challenge of describing the* 0^+_2 *resonance can be addressed within the current framework of nuclear Hamiltonians, without requiring modifications or extensions.*

   This conclusion is premature; see Comment 4 and notice that the resonance energy, width, and diffraction pattern are not reproduced. Also, the usage of "the current framework of nuclear Hamiltonians" is misleading, as the coupled-channel reaction calculations in this paper are based on the schematic nuclear potential, and the ab-initio input (nucleonic densities) is not discriminating.

10. *Indeed, establishing a transparent link between the measured cross section in an open quantum system, such as alpha-alpha scattering, and the resonance in one of its subsystems, remains an unresolved problem in quantum mechanics.*

    Authors seem to have overlooked the efforts to reconcile the shell model with nuclear reactions in both the real-energy and complex-energy formulations of the continuum shell model, where the relation between cross-section and poles of the scattering matrix has been extensively discussed.

Extended Data:

11. How do the theoretical densities presented in Figs. 6(a) and (b) compare to experimental data from electron scattering?.

Section 5.2.2:

12. *Ideally, both the interference with non-resonant continuum and the threshold effect should be included in the resonance line shape analysis. However, to our knowledge such a theoretical framework is not available to data and approximate schemes are needed.*

    Both effects find a natural description in the continuum shell model.

Section 7.1:

13. The coupled-channel analysis is based on the folded-potential approach with the BNP nuclear potential of Ref. [65]. It is a reasonable phenomenological tool, used previously to describe the $\alpha + \alpha$ scattering. But it is not state-of-the-art, and it has little to do with ab-initio coupled-channel theory, see the recent references mentioned above. As seen in Fig. 6, the model dependence of nucleonic densities is minor. How can the experimental results obtained in this work be thus used to discriminate between ab-initio models?

    To call the reaction theory analysis in this paper microscopic is an overstatement. The calculation is using the optical potential to determine normalization factors multiplying nuclear potentials (eq. 4 in Sec. 7.1) in the diagonal part of ISI and FSI interactions. The nuclear potentials (eq. 5) are calculated by double-folding the phenomenological BNP interaction. The assumption that the matter density is twice the charge density is reasonable for the ground state. Still, for the 0^+_2 state, the proximity of the nearby reaction thresholds may change this relation. One may go on and on listing conceptual limitations of the reaction calculations in this paper.

    One may consider the present calculation adequate in view of the problems with determining microscopically the ab initio optical potentials. However, it is still a long way to go.

Section 7.2:

14. The historical account is fair but misleading regarding Michel's work [72]. The Authors complain that Ref. [19,72] neglected three-body forces. We first note that 3N forces were not considered in the reaction calculations of Cappuzzello et al. Second, one cannot make a blanket statement about the impact of 3N forces without reference to the NN channel and the underlying Hilbert space. See, e.g., very relevant Fig. 3 and related discussion of Kravvaris et al. on the α–α scattering phase shifts, in which the authors conclude that the choice of 3N-force regulator plays a significant role in determining the behavior of the α–α scattering. Interestingly, their results based on the NN chiral interaction are very close to those obtained with $NN+NN_{lnl}$, which describe the scattering data well. Also, the quoted "optimal resonance energy" of Ref. [72] is in fact the real energy of the Gamow pole, not the real-energy fit to the Fano form factor. As stated above, these two definitions should not be confused.

15. The normalization of the continuum density discussed below Eq. (16) is misleading as it assumes that the stationary real-energy approach is the correct way of describing broad decaying states.

16. *In the range of energies probed in our experiment, the S-wave p + 3H phase shift δ(E) has a typical resonance behavior, namely a sharp increase reaching the value of 90 deg. In such a case, ΨE tends to have the form of a "quasi-bound" state, namely to be very large in the region where the four nucleons are close to each other, much larger than ΨA.*

    Such a behavior is not obvious in Fig. 7.

Summary

The new data probing the ^{3}He+n and ^{3}H+p continuum structure of ^{4}He are interesting and, in principle, important. The theoretical discussion, based on a traditional approach, is sound, but it has limited predictive power. The paper is inconclusive, and some discussion is overstated.
